# Ingredients, Processing, and Fermentation: Addressing the Organoleptic Boundaries of Plant-Based Dairy Analogues

**DOI:** 10.3390/foods11060875

**Published:** 2022-03-18

**Authors:** Aileen Pua, Vivien Chia Yen Tang, Rui Min Vivian Goh, Jingcan Sun, Benjamin Lassabliere, Shao Quan Liu

**Affiliations:** 1 Department of Food Science and Technology, National University of Singapore, S14 Level 5, Science Drive 2, Singapore 117542, Singapore; aileenpua@u.nus.edu; 2 Mane SEA PTE Ltd., 3 Biopolis Drive, #07-17/18/19 Synapse, Singapore 138623, Singapore; vivientang95@gmail.com (V.C.Y.T.); vivian.goh@mane.com (R.M.V.G.); jingcan.sun@mane.com (J.S.); benjamin.lassabliere@mane.com (B.L.)

**Keywords:** plant-based fermented foods, dairy analogues, flavour, organoleptics, functionality, biotransformation

## Abstract

Consumer interest and research in plant-based dairy analogues has been growing in recent years because of increasingly negative implications of animal-derived products on human health, animal wellbeing, and the environment. However, plant-based dairy analogues face many challenges in mimicking the organoleptic properties of dairy products due to their undesirable off-flavours and textures. This article thus reviews fermentation as a viable pathway to developing clean-label plant-based dairy analogues with satisfactory consumer acceptability. Discussions on complementary strategies such as raw material selection and extraction technologies are also included. An overview of plant raw materials with the potential to be applied in dairy analogues is first discussed, followed by a review of the processing steps and innovative techniques required to transform these plant raw materials into functional ingredients such as plant-based aqueous extracts or flours for subsequent fermentation. Finally, the various fermentation (bacterial, yeast, and fungal) methodologies applied for the improvement of texture and other sensory qualities of plant-based dairy analogues are covered. Concerted research efforts would be required in the future to tailor and optimise the presented wide diversity of options to produce plant-based fermented dairy analogues that are both delicious and nutritionally adequate.

## 1. Introduction

Dairy remains a relevant and major agricultural product, with global milk production hitting 861 Mt in 2020 and projected to grow at 1.7% p.a. to 1020 Mt by 2030 [1]. The international demand for traditional bovine dairy remains high and stable, as shown from the minimal impact of COVID-19 on dairy production [1]. Dairy products, in particular, have played an enduring and important role in the diet of the general population, where they are consumed not only for enjoyment but also for nutritional needs and specific health benefits such as probiotic intake. Such dairy products include but are not limited to fermented foods such as yoghurt, cheese, and kefir [2]. Despite the existing popularity of conventional bovine dairy products, consumers are beginning to actively seek alternatives to them due to their potentially long-term negative impact on human health and the environment, and other ethical implications [3]. Consequently, this has led to an increased interest in plant-based dairy substitutes, which are perceived to overcome the limitations of these traditional dairy products.

Plant-based dairy substitutes are of particular interest due to their added health benefits and have been growing in popularity and market size [4]. The plant-based dairy sector is expected to expand at a CAGR of 12.5% and reach a global market size of USD 52.58 billion by 2028 [5]. Compared to conventional dairy products, plant-based dairy substitutes offer many attractive features to consumers, including: “free-from” properties [6] for lactose, cholesterol, and dairy allergens such as casein [4,7]; reductions in consumer concerns about hormones and antibiotic residues; vegan-friendly labels (depending on additives) [3,7,8]; typically high content in vitamins, minerals, other bioactives, phytochemicals, and added functionalities, such as dietary fibre or pre-/probiotic activity [4,8]. The growing popularity of this market has resulted in an expanding range of dairy substitutes involving a huge variety of plant matrices [4,9]. These plant-based dairy substitutes can be broadly divided into two categories: dairy alternatives, where plant-based ingredients merely assume the role of a replacement carrier for nutrients and probiotics; and dairy analogues, where plant-based materials are transformed to recreate the flavour, texture, appearance, and often nutrition of real dairy products [10,11]. Some examples of dairy alternatives include fortified aqueous extracts or dairy probiotic-enriched juices, while milks, yoghurts and cheeses are popular mimics for dairy analogues. For the purposes of this review, we will focus on plant-based dairy analogues, which are modelled after specific dairy benchmarks.

Plant-based dairy analogues face stiff competition from traditional bovine dairy products in terms of flavour, functionality, nutrition, and cost [3,9], which can be challenging given the compositional constraints of the plant matrix. In addition, many of these products are formulated with significant proportions of fats, starches, and additives (flavourings, colourings, and stabilisers), which are perceived to be less nutritious than their equivalent bovine dairy products [12]. The functionalities of these plant-based analogues, e.g., their behaviour under high temperature, gel stability, or probiotic activity may also fall short of consumer expectations, and the analogues may require further restructuring to meet textural demands [9]. Currently, product formulation is highly dependent on additive use to achieve reasonable consumer acceptability of plant-based dairy analogues. However, this is antagonistic towards the simultaneous consumer desire for clean-label products [9]. Thus, it is of great research interest to develop means to emulate the organoleptic properties of conventional dairy products in plant-based analogues with minimal additive use. Therefore, fermentation has been a key focal point in recent years as a solution to creating a novel, clean-label dairy analogue.

Fermentation is traditionally or biochemically defined as the process by which microorganisms obtain energy in the absence of oxygen. In the context of fermented foods, however, it refers to a general process in which a food matrix is metabolised/ transformed by microorganisms to impart desirable properties. This typically includes improvements to flavour, texture, nutritional composition, and/or food safety of the final product [2]. Considering that many plant-based matrices have undesirable off-flavours and struggle to achieve a natural dairy-like profile without the extensive use of processing aids and flavourings, the incorporation of a fermentation process could improve the organoleptic properties of such plant-based products and develop a clean-label fermented dairy analogue with adequate consumer acceptability.

Therefore, it is the objective of this review to explore the ways by which fermentation may aid in the creation of an organoleptically satisfactory plant-based dairy analogue. At the same time, we recognise the synergistic potential of applying complementary strategies, such as raw material selection and other processing techniques, which can significantly enhance the quality of the end product. We have, thus, structured this review to achieve the three following goals:

1. To summarise the range of raw materials used to produce dairy analogues as well as relevant physicochemical properties that support their use in such formulations;
2. To present an overview of extraction and processing strategies to optimise these raw materials for fermentation and/or subsequent formulation of dairy analogues, with an emphasis on recent innovations (published work from 2012 to date);
3. To discuss the impact of fermentation on the organoleptic quality of plant-based dairy analogues, with examples from both the literature (again focusing on research in the past ten years) and the market.

## 2. Common Raw Materials for Plant-Based Dairy Analogues

Various raw materials of plant origin have been utilised for the production of fermented dairy analogues and alternatives. Dairy analogues are typically made from plant materials with higher protein and/or fat content as these two components are the most essential contributors to the texture and flavour of dairy analogues [13,14]. Proteins are responsible for many physicochemical properties relevant to dairy products such as water-holding capacity, gelation, gel strength, as well as the generation of flavour precursors and/or compounds. Fats, on the other hand, affect both mechanical and sensorial properties, including mouthfeel, flavour, and flavour-carrying capacity [10,13]. Consequently, most raw materials used for dairy analogue production tend to fall into one of the four following botanical classes: legumes; grains; nuts, drupes, and seeds; tubers. Most of these materials have the added benefits of being nutrient-dense, while tubers are noted for their low cost and relative underutilisation [15,16].

The aqueous extracts of these raw materials are typically labelled as plant-based ‘milks’ (this term is misleading since it is not comparable to mammalian milks) [12,17]. Therefore, we will refer to these ‘milks’ as plant-based aqueous extracts (PBAEs) in text. With the right extraction and processing techniques, these PBAEs can be treated as dairy analogues, and further fermented and/or formulated into fermented dairy analogues such as yoghurts and cheeses [12,18]. In this section, a brief overview of the four main botanical classes of plant-based raw materials used in dairy analogue production (legumes; grains; nuts, drupes, and seeds; tubers) is provided. Selected examples of raw materials used in dairy substitute production are also included, in view of their potential to be developed into dairy analogues. The carbohydrate, fibre, total protein, and total fat contents of these raw materials are summarised in Table 1.

### 2.1. Legumes

Legumes belong to the Fabaceae family, one of the most economically significant families of flowering plants. The legume refers to the fruit of the plants in the Fabaceae family, while the seeds within the fruit pods are typically called pulses. They are popular for their low cost compared to other staple food sources such as nuts, as well as their high protein content [22,23]. This makes them a prime choice for dairy analogues that require sufficient protein levels to meet both functionality and nutritional standards [18,22,24]. Legumes are also a good source of minerals and water-soluble vitamins, and they contain polyphenols and phytosterols, both of which are bioactive classes of compounds and provide various health benefits such as antioxidant properties and reduced cholesterol absorption [4].

Soybean (*Glycine max*) is the most well-known and consumed legume worldwide. The water extract of soybean is one of the most popular milk substitutes worldwide [4,25], and it is commonly used in cheese and yoghurt analogues [26,27,28,29]. With a wealth of research tying its consumption to anticancer and antioxidant activity, soy PBAE and its dairy analogues continue to be a nutritionally attractive plant-based alternative to cow’s milk despite lacking several nutrients such as calcium and vitamin B12 [25,30].

The native ‘grassy’ or ‘beany’ flavour in soy caused by hexanal and other aldehydic lipid oxidation (lipoxygenase, or LOX-catalysed) products, however, faces significant acceptability issues in some countries and is perceived as an off-flavour in dairy analogues [30,31,32]. Fermentation of soy PBAE using various types of lactic acid bacteria (LAB) has thus been extensively researched as a solution to enhance its nutrient content and sensory attributes, with additional benefits such as shelf-life enhancement [33,34].

Other types of legumes with similarly high dietary fibre content are also routinely consumed as part of the diet and used for dairy alternatives and analogues. While they may possess a lower nutritional content compared to soy, these legumes present a lower prevalence of allergenicity in the wider population than soy [23,24,35,36]. Some examples include chickpea (*Cicer arietinum*), which is the third most important legume worldwide [36,37]. Chickpeas are frequently consumed for their high nutrient bioavailability [38] and have been proposed as a suitable alternative to soy in plant-based dairy substitutes with or without fermentation [36]. Pea *(Pisum sativum*), lupin (*Lupinus albus* L.), lentil (*Lens culinaris*), fava bean (*Vicia faba*), and common beans (*Phaseolus vulgaris*) are also other legumes that have been applied in dairy substitute production [39,40,41]. While these legumes possess excellent functional and nutritional qualities, their lipid content and LOX-catalysed degradation of polyunsaturated fatty acids are believed to be a major cause of undesirable off-flavours [42]. One legume of note is the peanut (*Arachis hypogaea* L.). Although it is scientifically known as a legume, it has a composition closer to that of nuts, drupes, and seeds. The peanut is valued for being nutritious yet affordable with a pleasant taste, although it can be highly allergenic like soy [43]. However, its composition and minimal off-flavour still makes it attractive for use in dairy analogue production.

### 2.2. Grains (Cereals and Pseudocereals)

Grains, which includes cereals and pseudocereals [17,20], typically make up a large part of the staple diet in many countries due to their low cost and high nutritional content. Grains such as oat (*Avena sativa*) provide a mixture of nutrients such as carbohydrates, dietary fibre, and minerals, with some studies also reporting appreciable antioxidant and prebiotic properties [44]. Compared to other grains, oat contains a higher amount of lipids, which is a desirable trait for mimicking the mouthfeel of dairy analogues [45]. Oat is also reputed to be a great source of dietary fibre due to their high *β*-D-glucan content, which has multiple health benefits such as lowering blood glucose and cholesterol levels [10,45,46].

Rice (*Oryza sativa*), being the most popular staple grain, has also seen some uses in the formulation of dairy alternatives. Notably, rice bran, which is a by-product of rice during the milling process, has been increasingly explored due to its nutrient content and underutilisation [47]. Other grains applied in or potentially useful for the production of dairy analogues include quinoa (*Chenopodium quinoa*), a vitamin-rich pseudocereal containing all essential amino acids [44,48,49], and barley (*Hordeum vulgare*). Barley, in particular, is a carbohydrate- and fibre-rich material that is suitable for food fermentation applications. Despite the abundance of barley (accounting for more than 10% of all grains grown worldwide), less than 20% of the harvested crop is used for human consumption [15]. Collectively, grains remain underutilised as a nutritious raw material for dairy analogue production despite their mild flavour [15,47], which is a highly attractive trait for mimicking the flavour of conventional dairy; therefore, they deserve a greater amount of research interest.

### 2.3. Nuts, Drupes and Seeds

Nuts, drupes, and seeds are renowned for their high protein, minerals, and vitamin E content, the latter of which is known to exhibit antioxidant properties. In addition, they also contain phytosterols that reduce dietary cholesterol absorption [50,51]. Notably, their fat content ranks among the highest of all plant-based food materials. The majority of lipids found in nuts, drupes, and seeds are unsaturated fatty acids, which are beneficial to health [19]. In addition to their nutrient content, this class of raw materials has an advantage in consumer acceptability over other raw materials due to their natural nutty flavour, which is more compatible with conventional dairy flavour compared to the beany, earthy flavour of legumes and grains [52,53].

Two of the most popular dairy substitutes from this category are derived from almond (*Prunus amygdalus*) and coconut (*Cocos nucifera*). Compared to other types of PBAE, almond PBAE has a naturally creamy texture, which makes it suitable as an analogue for cow’s milk and its derived products [52,54,55]. While almond PBAE is more widely consumed in North America and Europe, coconut PBAE is more frequently found in Southeast Asian countries [56,57]. Coconut PBAE has a major advantage over nut PBAEs as a raw material for dairy analogues because they do not contain typical nut allergens [57]. In general, their uniquely high fat and protein content have led to the frequent application of nuts, drupes, and seeds as raw materials in dairy analogue production [13].

### 2.4. Tubers

Similar to grains, tubers are another globally consumed staple crop, with the most popular tuber being the potato. These underground roots are prized for their resistance against adverse weather conditions such as drought, and they are utilised for a variety of applications [58]. Other tubers such as yam (*Dioscorea* spp.) and cassava (*Manihot esculenta*) have been growing in popularity outside of countries where they are consumed as a staple food. In addition to being rich in carbohydrates, yam contains many bioactive compounds and has been used in traditional remedies [16,59,60]. A noteworthy study by Batista et al. explored the use of yam dough in ice cream making as a substitute for cow’s milk [61]. While yam is not known to be fermented for direct consumption, more studies have emerged, showing an improvement in the nutritional and sensorial aspects of this relatively underutilised crop via fermentation [16].

While various cassava snacks can be found on supermarket shelves worldwide, this crop remains most relevant in sub-Saharan Africa [21]. This is due to several properties of cassava that allow it to become a crop with high food security: the resistance of cassava roots to various pests and undesirable growing conditions; staggered harvesting as they can remain in the ground for a long time; and the highest calorie-to-cost-and-space ratio among other crops [21,60]. Additionally, a variety of fermented cascaras are consumed as staple foods in Africa, and the diversity of fermentation styles induces variations in its sensory aspects and contributes to food preservation and nutritional enhancement [58]. Despite this, cassava has not quite been explored as a dairy analogue ingredient beyond only a very few publications [62].

The tiger nut (*Cyperus esculentus*), a lesser-known tuber grown in Spain and Western African regions, has also been gaining increased global attention. Consumed frequently in West Africa due to its low cost and availability, the tiger nut plant is considered a weed due to its invasive nature [8,63]. However, compared to other tubers, the tiger nut has higher dietary fibre and protein content [8,51]. As such, there is increasing research interest in the traditional tiger nut ‘milk’ (or beverage), which is obtained by soaking, grinding, and pressing dried tiger nuts, and it has been compared to other mainstream PBAEs such as almond or soy PBAEs [8,64]. With appropriate processing and formulation strategies, high food security crops such as tubers could offer a unique advantage as cheap raw materials for dairy analogue production.

### 2.5. Challenges of Producing Plant-Based Dairy Analogues

Due to the vast differences in the chemical compositions between plant and dairy raw materials, mimicking both the nutrition and sensory profile of conventional dairy products continues to be a key challenge in the creation of dairy analogues. Additionally, though soy and other legumes have been used extensively as milk alternatives or analogues, their naturally ‘beany’ odour is perceived as an off-flavour in the context of dairy profiles [4]. These raw materials may also contain antinutrients such as inositol phosphate, which reduces the nutritional quality of the plant-based products by impeding the absorption of nutrients such as minerals [44,45].

The shortfalls of plant-based dairy analogues versus their dairy counterparts have often been addressed by the addition of stabilisers, fillers, nutrients, and other processing aids [32,45,65]. For example, oils are commonly added for flavour and texture purposes and lecithin for emulsion stabilisation [4]. Plant-based dairy analogues also tend to be fortified with vitamins (e.g., A and D) and minerals (e.g., calcium) to nutritionally resemble bovine milk [4]. However, this may decrease their appeal to consumers due to increasing demands for clean-label products and the known flavour and nutritional issues with ultra-processed plant-based foods [10,66]. Consequently, research has turned to the use of fermentation to narrow the gap between dairy analogues and their benchmarks without excessive additive use. Numerous studies have demonstrated the positive impact of fermentation on the nutritional and organoleptic qualities of plant-based dairy analogues, including but not limited to the degradation of antinutrients, probiotic function, textural improvement, and off-flavour reduction [4,37,47,65]. These will be covered in detail in Section 4 of this review.

Nonetheless, the application of appropriate raw material extraction and processing strategies is warranted to complement and amplify the positive impacts of fermentation on the organoleptic quality of plant-based dairy analogues. These techniques comprise a range of mechanical, chemical, biological, and novel processing methods, with a common aim of obtaining a matrix with the best functional properties for the subsequent production of dairy analogues. The next section of our review provides insights into recent studies on these extraction and processing strategies, with a key focus on organoleptic improvement.

## 3. Extraction and Functionalisation of Ingredients from Plant Raw Materials

**Figure 1:**
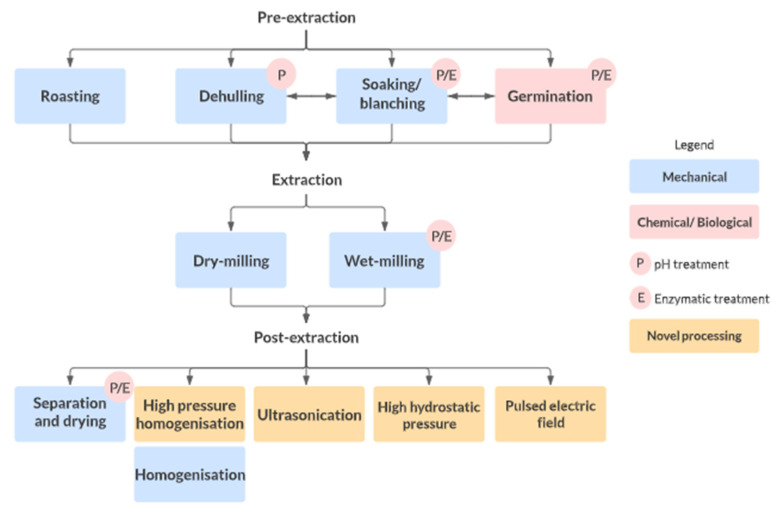
Overview of possible processing techniques for plant-based ingredient extraction for dairy analogue production.

Aside from the selection of raw materials with suitable flavour and nutritional properties, the extraction of such plant-based materials to generate suitable ingredients is also critical to the development of a satisfactory plant-based dairy analogue. Extraction processes have a profound effect on the composition of the raw material, which then determines its behaviour during subsequent product development stages [7]. As discussed in Section 2, the extraction and characteristics of fat and protein are critical considerations in the production of dairy analogues due to their effect on the ingredient’s functional properties and flavour [67]. In particular, extraction treatments as well as the relevant pre- and post-extraction treatments (depicted in Figure 1) can lead to varied ingredient microextractions and protein conformations. These can affect solubility, water absorption capacity, gelation, and emulsion stability, all of which ultimately modify the end product texture [67]. Based on the organoleptic limitations of plant-based raw materials (Section 2), considerations regarding the generation of flavours and reduction or elimination of off-flavours are also warranted [17]. These organoleptic qualities are the main determinants of product acceptability and, hence, are the focus of this discussion, though we will provide a brief discussion on the effect of these extraction techniques on the recovery of some chemicals of health interest [68]. A summary of these studies is provided in Table 2.

In the process of creating a dairy analogue, two main ingredient classes derived from plants are considered: solid flour extracts and PBAEs. PBAEs tend to be the ingredient of choice for the development of dairy analogues and will be reviewed in greater depth. It is also worth noting that different plant raw materials can exhibit various trends upon treatment, and these differences are discussed to provide an overview of the diversity of manipulations and their effects on ingredient quality [9].

### 3.1. Conventional Mechanical Operations

For most studies, the mechanical extraction of plant raw material is applied. Mechanical treatments allow for standardisation of the process and are effective in dispersing the raw material for ingredient extraction. In addition, they are low cost, scalable, and have a low technological barrier [7,17]. Along with these mechanical operations, thermal treatments may be conducted at several points to improve extraction yield or ingredient properties.

#### 3.1.1. Pre-Treatments

*Roasting—*Prior to milling, a variety of pre-treatments may be conducted. Thermal treatment with roasting has been applied to specific raw materials (Table 2) such as legumes and peanuts to enhance the flavour and aroma of their extracted flours and PBAEs [17,67,69,70] and was also noted to reduce LOX-generated off-flavours in sesame [70]. The sensorial acceptability for such thermally generated flavours (‘roast’ notes) in dairy analogues, however, requires further study. Roasting also contributes to protein denaturation, which alters the ingredient’s functionality [7]. It reduced protein solubility for sesame PBAEs [70] but conversely increased protein solubility and emulsion stability for peanut PBAEs [69]. More studies on the impact of roasting parameters and their variation with raw materials are needed for a better understanding of its influence on protein functionality.

*Dehulling—*Dehulling is suitable for isolating desired elements from raw materials that possess numerous components (e.g., legumes and grains) [23] (Table 2). Both dry-dehulling and wet-dehulling may be conducted, although there are few studies observing the impact of this selection on the functionality and flavour of plant-based dairy analogues. Dehulling removes dietary fibres as demonstrated in several legume flours [72], which may result in smoother, less gritty, and more pleasant textures in dairy analogues. The resulting concentration of the endosperm may also remove off-flavours and antinutrients for a variety of legumes [72,100]. However, the impact of dehulling depends on downstream processes such as soaking. Ma et al. demonstrated that dehulled peas potentially released more ‘small molecules’ during soaking, which resulted in lower amounts of the off-odorant 2-methoxy-3-isopropyl-(5/6)-methyl pyrazine and albumin (which did not negatively impact texture in this study) in the obtained pea PBAE [71]. It is thus evident that the effects of pre-extraction treatments can be interdependent.

*Soaking and blanching—*The raw plant material may be either soaked in cold water or blanched in hot water to soften it and remove undesirable water-soluble components [9,17,75] (Table 2). While the additional heat from blanching can result in more efficient removal or inactivation of undesirable off-flavours and antinutrients compared to soaking, it can also affect the functionality, flavour, and nutritional value of the final ingredient [12]. For example, flours from boiled and roasted pulses (seeds of legumes) displayed 2–3 times higher water absorption capacities and higher gelation rates than flours from raw pulses [67]. Heat treatment from blanching was also found to inactivate undesirable endogenous enzymes to further improve flavour and nutrition [9], such as LOX that produce off-flavours in soy and peanut PBAEs, and trypsin or other protease inhibitors that restrict protein digestion [7,67,101]. However, blanching can also result in the loss of desirable nutrients such as proteins, choline, and folate, although the extent of such losses is dependent on the raw material and temperature used [67,101]. While the impact on functionality and nutrient composition can be quite varied, overall, soaking, and especially blanching, are effective methods for off-flavour removal.

#### 3.1.2. Extraction and Separation

Mechanical grinding is then performed on the untreated or pre-treated plant tissue [9]. For flour ingredients, dry-milling is usually first conducted, and the flour would later be reconstituted or further extracted to create the final product [17]. For PBAE ingredients, aqueous extraction is required, and the raw material is typically ground into a slurry by wet-milling to release soluble or finely suspended materials [7,17]. Both the resulting flours and PBAEs possess a non-homogenous particle size distribution that may require further size standardisation or reduction for texture and stability [64]. Superfine pulverisation technologies have been gaining attention for plant flour ingredients such as colloid-milling, jet-milling, and ball-milling [23,102]. Related to this point, homogenisation for PBAE ingredients is discussed in Section 3.3.1.

Conventional milling processes can be innovated by tailoring parameters such as oxygen availability and temperature, which affect the composition and organoleptic qualities of the extracted plant ingredient. Kaharso et al. recently investigated anaerobic wet-milling of soy with oxygen-free water and found that it significantly reduced the formation of lipid oxidation products and off-odorants (e.g., alcohols and aldehydes) in the resulting soy PBAE [74]. Thermal treatments are also commonly applied for wet-milling (e.g., cooking the slurry) to increase extractability [7,17]. However, the impact of thermal treatment is not straightforward. For example, high temperature cooking increases nutrient solubility and recovery, but extreme temperatures can denature plant proteins and decrease their yield and/or alter functionality [7,103]. Thermal treatment during or directly after milling makes a significant contribution towards the deactivation of endogenous enzymes to reduce off-flavour production and antinutrient content, especially for soy [103]. High temperature treatment can also increase oil extractability [7,17] and affect starch gelatinisation [7,13]. These effects on downstream processing and organoleptic qualities on oil-rich nuts or starch-rich grains can be positive, such as the generation of desirable flavours and textures (e.g., in yoghurts), or negative, where extra processing steps may be required.

Finally, separation of the unwanted material (typically coarse particles in the PBAE) or concentration of desirable components (e.g., proteins) occurs by decanting, gravity, centrifugation, or (ultra)filtration [9,104] to remove excess lipid materials and prevent the coalescence of oil bodies and phase separation, which ensures product stability and a consistent lipid proportion in the ingredient [54,65]. If necessary, a final drying step (e.g., spray-drying) may also occur for easier transportation or incorporation of the completed ingredient.

### 3.2. Chemical and Biological Aids

Although mechanical methods form the backbone of raw material isolation and functionalisation strategies for flour or PBAE creation [17], there has been increased incorporation of chemical and biological techniques to improve the resulting ingredient quality for flavour or fermentation purposes [9]. Off-flavours remain a major concern for plant-based ingredients as these significantly hinder their applicability to bovine-milk-based products, especially fermented products such as yoghurt and cheese, as consumers are sensitive to the typically ‘grassy’ or ‘earthy’ off-flavours [81]. A variety of chemical and biological techniques have thus been applied to strategically deodorise the ingredients yielded from plant raw materials or to functionalise other components.

#### 3.2.1. pH Treatment and Other Chemical Extraction Techniques

*pH alteration*—The pH of the soaking or extraction environment has commonly been altered to facilitate protein extractability, especially to manipulate the solubility of proteins based on their isoelectric points, which may be acidic or alkaline depending on the raw material [17,70,75] (Table 2). In cases where powdered protein isolates are the target ingredient, pH alteration could be utilised for isoelectric precipitation to induce protein aggregation for subsequent extraction [4,13]. More interestingly, pH alterations can also be applied to reduce off-flavour formation. Ahmadian-Kouchaksaraei et al. reported higher LOX activity of sesame seeds in acidic conditions (pH 5) [70], and it is a common industrial practice to alkalinise soy or peanut PBAEs with sodium bicarbonate to reduce LOX activity and lipid oxidation off-flavours [70,105]. pH also affects protein solubility and functionality [75] and was manipulated by Ma et al. to alter gel hardness for pea yoghurt, which resulted in softer and more sensorially acceptable yoghurts [71].

*Chemical and physical deodorisation—*Such techniques are largely used for plant protein isolates or plant flour ingredients, and despite their promise for off-flavour reduction, have not been directly assessed for use in plant-based dairy analogues [76,77,78] (Table 2). Wang et al. demonstrated that alcohol washing of pea protein flour resulted in deodorisation and the removal of off-flavours, though high alcohol washes led to reduced emulsion stability and solubility of the pea flours [78]. Sorptive techniques are less common but also effective for off-flavour removal. For example, polystyrene and zeolite-based adsorbents were applied to remove the majority (60–70%) of hexanal from soy protein isolate [31]. Most commonly, vacuum treatment at high temperatures has been applied to soy and peanut PBAEs to indiscriminately strip it of its characteristic aromas [105,106] that would be unpleasant in the context of dairy products. Distillation techniques (physical deodorisation) such as supercritical carbon dioxide extraction have been effective in removing off-flavours from pea flours [76] (Table 2). As these chemical methods (along with solvent and sorptive extractions) effectively strip the raw material of most odorants, the aroma would have to be reintroduced to the product through other ingredients or fermentation strategies.

#### 3.2.2. Enzymatic Treatments

The variety of commercially available food-grade enzymes has vastly improved the quality of plant-based ingredients. Plant materials differ from bovine milk due to the presence of fibre as well as large oligomeric proteins, which results in gritty textures [81] and reduced emulsion stability, leading to undesirable mouthfeel in dairy analogues [7]. The majority of the enzymes applied to plant-based dairy analogues are used for the hydrolysis of macromolecules to reduce particle size and improve solubility and mouthfeel [81]. Li et al. demonstrated that papain treatment in soy cheese was able to hydrolyse proteins and yield a more homogenous protein network with improved textural and hedonistic qualities [81]. Luana et al. also demonstrated that enzymatic (Depol 740 L and Grindamyl 1000) treatment of an oat yoghurt beverage during fermentation led to improved aroma and taste characteristics as well as reduced fermentation latency, possibly due to its release of free sugars and other fermentation substrates [53]. As such, enzymatic treatment is especially complementary to the fermentation process in improving end product organoleptics.

Another major application for enzymes in PBAE ingredients is starch liquefaction (as mentioned in Section 3.1.2), typically with *α*/*β*-amylases, to reduce the viscosity and improve the fluidity and emulsion stability of the PBAE [14,19], which may also be beneficial for releasing free sugars for subsequent fermentation. While enzymes are a powerful tool to improve the functionality of plant-based dairy ingredients and analogues, they are highly specific and costly. Where the use of multiple enzymes is warranted, fermentation may prove to be a cheaper and more sustainable alternative.

#### 3.2.3. Sprouting and Germination

Another cost-effective alternative to enzymatic treatment is the natural germination of the plant raw material. During germination, proteases and amylases among other enzymes are activated, which can significantly alter the composition and functional properties of the plant material [4,82]. Decreases in the water-holding capacity of soy yoghurts were observed with germination (and degree of germination), likely due to starch hydrolysis, which altered the gelation properties of soy PBAE and yoghurt, resulting in more sensorially acceptable textures. Germination has also been reported to improve the flavour characteristics and sensory acceptance of rice and soy yoghurts [83,84,85] (although the opposite was reported for sprouted tiger nut yoghurt [82]), due in part to a reduction in LOX activity with germination, which is a trend observed in PBAEs [24]. Germination-derived increases in free sugars and amino acids may serve as valuable precursors for the formation of pleasant aroma compounds during fermentation [85]. This also explains the observation that germinated plant material improves the growth kinetics of starter cultures during fermentation, which synergises with the application of fermentation for the organoleptic improvement of plant-based dairy analogues [83].

Aside from texture and flavour benefits, germination was found to improve the nutritional properties of fermented plant-based dairy analogues by decreasing the antinutrient content or increasing the amount of *γ*-aminobutyric acid owing to increased glutamic acid decarboxylase activity [82,84]. For studies that focused on the extraction of PBAEs, germination was also observed to increase the ingredient’s antioxidant activity and decrease antinutritive content (e.g., saponins, phytate, trypsin inhibitors) depending on the germination duration (Table 2) [4,24,107,108]. As germinated plant ingredients are viewed by consumers to be nutritionally superior and natural, germination appears to be extremely viable for improving ingredient and fermented product quality.

### 3.3. Enhanced Functionality with Innovative Processing

For plant-based ingredients to be made into dairy analogues, the main challenges are the texture, off-flavour, and antinutrient content [47,109]. When it comes to texture and stability, PBAEs possess thermodynamically unstable and polydisperse particle distributions due to their diversity of particles (e.g., oil droplets, native protein aggregates, polysaccharides, and cell fragments) [4,110]. This makes PBAEs especially prone to sedimentation, creaming, or syneresis over storage [4,109,110], which complicates their application as ingredients for dairy analogues. Most conventional mechanical or chemical processing methods do not address this aspect of the plant material microstructure. Hence, greater interest has arisen in innovative processing technologies, and some achievements and limitations are discussed here.

#### 3.3.1. Homogenisation by HPH and Ultra-HPH (UHPH)

Homogenisation has a huge impact on the extracted PBAEs’ microstructure [111] and is also often applied to bovine milk for texture and stability improvement through the reduction and standardisation of the size of fat globules [9,54]. Due to the different microstructures and constituents of plant-based ingredients, conventional homogenisation parameters for bovine milk are unlikely to perform equivalently well in PBAEs. The particle heterogeneity of PBAEs demands more aggressive processing parameters than those used in bovine milk (10–25 MPa) [109], and some studies have investigated the potential benefits of HPH and ultra-HPH (UHPH) to improve the stability and organoleptics of PBAE ingredients.

HPH and UHPH could assist in achieving artificial globules that resemble bovine milk viscosity, stability, and mouthfeel for organoleptic purposes [9] as well as modify protein conformation and functionality (e.g., emulsifying or foaming properties) for texture improvements. This particularly benefits yoghurt analogue production by creating PBAE ingredients with improved textural properties. Ferragut et al. demonstrated in soy-based yoghurts that UHPH (200–300 MPa) soy PBAE resulted in preferable textures compared to thermally treated soy PBAE. UHPH-treatment was found to increase the onset of gelation and decrease aggregation rate and gel network density, resulting in improved mechanical properties such as higher firmness, water-holding capacities, and more compact network structures in the soy yoghurt product [89,112,113]. Demirkesen et al. also demonstrated that the microfluidic treatment of hazelnuts (135 MPa) to create a whole hazelnut PBAE resulted in improved hazelnut yoghurt texture, which was closer to that of conventional yoghurt [88]. Generally, the application of HPH techniques to PBAEs resulted in highly stable emulsions with viscosities and mouthfeels similar to bovine milk (Table 2) [91], which makes HPH an attractive processing strategy in dairy analogue production [114].

Although less well studied, HPH could also improve the flavour of plant-based ingredients. Poliseli-Scopel et al. and Pérez-González et al. both studied changes in the volatile composition of soy and almond PBAEs, respectively, with UHPH compared to other processing methods [115,116]. They noted that UHPH-treated PBAEs tended to attain improved sensory characteristics, particularly with lower lipid oxidation markers, which may indicate lower off-flavour formation by LOX due to inactivation by the high shear forces (Table 2).

#### 3.3.2. Innovative Non-Thermal Technologies

Heating in the form of pasteurisation and ultra-high temperature (UHT) treatment is commonly applied for microbial inactivation in PBAE ingredients, though this can lead to the formation of thermal off-flavours in plant-based dairy analogues [68]. While many innovative non-thermal techniques have been assessed for microbial inactivation purposes [68], they may also play additional roles for texture and flavour stability. As such, modern techniques (e.g., ultrasonication, high hydrostatic pressure (HHP), and pulsed electric field (PEF) treatment) that have been used in plant-based dairy applications were evaluated.

*Ultrasonication—*Aside from HPH, ultrasonication has also been explored for the reduction and standardisation of particle sizes and for protein modification. Ultrasonication was found to reduce particle size and distribution in coconut PBAEs, resulting in greater stability and increased fluidity [93,94,117]. Depending on the ultrasound parameters, a variety of protein modifications may also result in changes to the ingredient’s textural functionality, as reviewed by Gharibzahedi et al. for legume proteins [118]. While ultrasonication appears to have large benefits in terms of ingredient texture, it tends to promote destructive oxidative processes [118], which may affect the flavour of plant-based dairy analogues.

*HHP—*For the creation of dairy analogues, HHP is of interest due to its impact on protein functionality. HHP was found to induce gelation with plant protein materials, which may be applicable for certain products such as yoghurts [96] and was successfully applied to increase water-holding capacity and reduce syneresis in soy yoghurts [95]. For materials where off-flavour production is enzymatic in nature, HHP may also improve sensory characteristics by enzyme inactivation, as demonstrated by its ability to reduce LOX activity in soy yoghurts and PBAEs resulting in fewer lipid oxidation off-odorants [95]. HHP has also been shown to reduce the allergenic characteristics of plant proteins from the raw material, which is beneficial for consumer health.

*PEF—*PEF involves placing the food product between two electrodes and subjecting them to short pulses at high voltages for a relatively short processing time (compared to thermal treatment). Thus far, PEF has been applied to PBAE ingredients and has mainly appeared to be effective for reducing PBAE particle size and increasing colloidal stability [119]. PEF may be similar to HHP [120] in that it deactivates enzymes that result in off-odour formation (Table 2) [98], showing potential for its use for dairy analogues.

#### 3.3.3. Blending Plant Materials and Additives

Ultimately, each raw material faces unique drawbacks for use in plant-based dairy analogues due to a mix of organoleptic and nutritional disadvantages that may be extremely challenging to evade even with sophisticated processing methods. Blending plant raw materials at different ratios has thus been of great interest to improve the flavour profiles, textures, and nutritional properties of dairy analogues [32]. Coda et al. blended cereal and soy flours with concentrated grape must to create yoghurt beverage analogues, and they found that mixtures of rice and barley or emmer flours resulted in improved organoleptic and nutritional properties compared to pure rice flour ferments [121]. Adejuyitan et al. found that a 50:50 soy/coconut PBAE-based cheese substitute yielded a higher hedonistic rating than a 100% soy version [122]. Additionally, Oyeyinka et al. demonstrated that a cheese analogue containing a 2:3 ratio of cashew to soy PBAE achieved the highest protein content and hedonistic rating for flavour [123]. While these studies showed that blending raw materials could result in improved hedonistic ratings, the mechanism behind this is unclear, although Short et al. suggests that one potential reason could be the masking of off-flavours, e.g., the beany note in soy [32]. Blending raw materials is also of great nutritional interest, especially for the amino acid profile of the ingredients. As discussed in Section 1, plant materials, unlike bovine dairy, have lesser amino acids and comparatively poorer digestibility [4]. Hence, purposeful blending of plant materials could help to achieve a more complete amino acid profile without fortification. In summary, blending different raw materials could result in an ingredient with improved properties for subsequent formulation or fermentation into a plant-based dairy analogue with improved sensorial and nutritional properties.

## 4. Fermentation as a Strategy to Improve Organoleptic Properties of Plant-Based Dairy Analogues

Collectively, the selection of appropriate raw materials, extraction, and processing strategies complements the use of fermentation in creating a plant-based dairy analogue with an authentic, dairy-like sensory profile. Fermentation can play a defining role in generating dairy-like flavour and textural attributes in plant-based dairy analogues. These include the modification of sensory characteristics such as acid production, masking or eliminating native off-flavours in plant materials, and secreting exopolysaccharides (EPS) that thicken the plant matrix to emulate the creamy texture of dairy products [13,124]. Fermentation has been shown to improve the nutritional content of plant-based dairy analogues by increasing the bioavailability of nutrients, reducing antinutritive components and/or allergens, as well as additional probiotic functions [4,125]. It also improves the safety and shelf life of these analogues through acidification, the generation of antimicrobial compounds, and competition with undesirable microorganisms [125,126,127].

There exists a multitude of starter cultures available to the researcher or manufacturer seeking to apply fermentation to the development of a plant-based dairy analogue. Table 3 summarises some examples of fermented plant-based dairy analogues and starter cultures that are available in the market. In the interests of enhancing the organoleptic properties of a dairy analogue, many studies and commercial products utilise starter cultures associated with traditional fermented dairy products. A brief overview of such cultures is given in the following sections before the provision of specific examples in different product categories (fermented cream products, yoghurt, cheese, kefir). Interestingly, the complexity of mimicking an authentic dairy profile—be it flavour, texture, or appearance-wise—may lead to the use of strains not usually associated with dairy fermentations [128].

### 4.1. Brief Overview of Microorganisms Involved in Traditional Dairy Fermentation

#### 4.1.1. Lactic Acid Bacteria

Of the array of microorganisms involved in food fermentation, lactic acid bacteria (LAB) are an indispensable group when it comes to dairy fermentation. They are a phylogenetically heterogenous group comprising Gram-positive, non-motile bacteria including the genera *Lactobacillus (L.)*, *Lactococcus (Lc.)*, *Leuconostoc (Leu.)*, and *Bifidobacterium (B.)*, to name a few. Different members of the LAB family may perform homolactic or heterolactic fermentation based on hexose catabolism and other environmental factors. Homolactic fermentation is common in LAB associated with meat and dairy fermentations where acidification is the primary function of fermentation, and the major end product is lactic acid. Heterolactic fermentation, on the other hand, tends to be more prevalent in plant-based fermentations compared to meat and dairy, where it generates significant amounts of acetic acid, ethanol, and CO_2_ along with lactic acid. Other metabolic processes relevant to the sensory aspects of fermented dairy include citrate utilisation that generates acetic acid, lactic acid, diacetyl, acetoin, and CO_2_ [129,130].

There have been several taxonomic changes to the lactobacillus group of late. However, they have yet to be validly published (considered as accepted, now published in [131] and being adopted) under the rules of the International Code of Nomenclature of Bacteria [131,132]. For the purposes of this review, we will continue using the old nomenclature of Lactobacillus for the following species: *Lactiplantibacillus plantarum*, *Lactiplantibacillus pentosus*, *Lacticaseibacillus casei*, *Lacticaseibacillus paracasei*, *Lacticaseibacillus rhamnosus*, *Limosilactobacillus fermentum*, *Limosilactobacillus pontis*, *Limosilactobacillus reuteri*, *Latilactobacillus curvatus*, *Fructilactobacillus sanfrancisensis*, *Levilactobacillus brevis*, *Liquorilactobacillus nagelii*, *Lentilactobacillus hilgardii*, *Lentilactobacillus buchneri*, and *Lentilactobacillus kefiri*.

In dairy fermentations, LAB play a crucial role in flavour formation, textural modification, and food preservation. The application of probiotic strains confers additional health benefits to the consumer [127,129,133]. Their safety and ubiquity are well-documented, making them a popular choice in fermentation studies, even if the raw material in question is not a native environment for LAB growth [4,134,135]. LAB often assume the role of starter cultures in dairy fermentations, e.g., *St. thermophilus* and *L*. *bulgaricus* in yoghurt, and *L. kefiranofaciens* and *L. kefiri* in milk kefir [133,136]. They are also used as adjunct cultures for organoleptic improvement, particularly in cheeses. These non-starter LAB (NSLAB) include strains such as *L*. *casei*, *Leu. mesenteroides*, *Leu. dextranicum*, and *L. plantarum* [137].

#### 4.1.2. Yeasts and Filamentous Fungi

In contrast, yeasts and fungi are seldom used as starter cultures in dairy fermentations. Many strains are associated with surface- and mould-ripened cheeses [138,139]. Yeast and fungi play several distinct roles in cheese fermentation. Some species ferment lactose, such as *Kluyveromyces lactis*, *K. marxianus* (*Candida kefyr*), and more rarely, *Saccharomyces cerevisiae*, while others assimilate lactose and/or galactose, e.g., *Debaryomyces hansenii*, *Geotrichum candidum*, and *Penicillium camemberti*. Additionally, *G. candidum*, *P. camemberti*, and some strains of *D. hansenii* assimilate lactate, raising the pH of the cheese matrix and hastening the process of ripening. Several species are especially prized for their strong proteolytic and lipolytic activities, including *G. candidum*, *Yarrowia lipolytica*, and several *Penicillium* spp. Their broad enzymatic activity liberates free amino acids and fatty acids in the matrix, consequently imparting the cheesy, umami, and bitter notes we associate with cheese [137,138,140,141]. Mould-ripened cheeses such as Brie, Camembert, and blue-veined cheeses owe their distinctive appearances and flavour to fungal adjunct cultures.

Spontaneous fermentations are responsible for the birth of fermented milk drinks, including kefir, koumiss, gariss, and chal. They are generally recognised to be the product of mixed culture, yeast-LAB fermentations, and their primary differences lie in microbiota composition and milk source [142,143,144]. Though the production of different fermented milks is traditionally restricted to specific regions and cultures, there is increasing interest in popularising such beverages for their purported health benefits [145,146]. Yeasts that ferment or assimilate lactose predominate in fermented milk matrices, including *C. kefyr*, *K. marxianus*, and *K. lactis*. All other associated yeast species generally metabolise lactate, usually under aerobic conditions, such as *Issatchenkia orientalis* (*Candida krusei*), *Y. lipolytica*, and *Saccharomyces unisporus*. Reminiscent of cheese fermentation, lactose-fermenting yeasts ferment the residual lactose in the medium and/or raise the pH through lactate assimilation. At the same time, they produce substantial quantities of ethanol and carbon dioxide, giving some fermented milks their signature fizzy and alcoholic flavour [144,147,148,149].

### 4.2. Fermented Plant-Based Dairy Analogues

The following subsections provide selected examples of studies performed in the last ten years (2012 to date) pertaining to the organoleptic improvement of plant-based dairy analogues via fermentation. These include the enhancement of flavour, texture, appearance, and/or other sensory characteristics to better mimic a defined dairy benchmark of the plant-based analogue. As such, several types of studies are excluded from this review, including but not limited to: (1) studies simply assessing probiotic viability in plant-based raw materials; (2) the application of classic dairy fermentation cultures and protocols (yoghurt, kefir, etc.) in plant matrices without evaluation of their organoleptic properties or comparison against a dairy benchmark; (3) nutritional studies on fermented, plant-based dairy analogues. A brief overview of the studies included is summarised in Table 4.

In literature, the fermentation of plant-based milk analogues is often designed with nutritional improvement in mind; many studies also evaluate probiotic viability [4,65]. Notably, a study by Tangyu et al. screened a number of microorganisms for their potential in enhancing the nutritional and sensory profile of chickpea-based milk analogues [37]. A significant decrease in off-flavour aldehydes was observed, accompanied by an increase in sweet, fruity, and creamy notes, especially with citrate supplementation. Commercially available plant-based milk analogues, however, generally rely on processing techniques and/or formulation strategies to mask unpleasant notes and resemble the organoleptic profile of dairy milk [4,65]. It is plausible that fermentation is rarely practiced for the organoleptic improvement of commercial milk analogues because it is less economically feasible than other processing strategies, or that it may result in acidification, which could curdle plant proteins and negatively affect the fluidity of the milk analogue [13].

#### 4.2.1. Fermented Cream Products

Butter (lactic butter), lactic or traditional buttermilk, and sour cream are all products of cream fermentation. To make lactic butter, cream—typically from cow’s milk—containing at least 40% fat is fermented with pure or mixed LAB cultures of *Lactococcus lactis* and *cremoris*, *Lc. lactis* biovar. *diacetylactis*, and *Leu. cremoris* or other diacetyl-producing LAB strains before churning; the liquid expelled during this process is known as buttermilk. Nowadays, it is more common for buttermilk to be made by directly fermenting semi-skimmed milk with the same strains used in butter fermentation. A similar process applies to the manufacture of sour cream, though no churning is involved [127].

At the time of writing, we had only identified one study on fermented plant-based cream products that fell within the scope of our review. Madsen et al. explored the possibility of producing a buttermilk koldskål (Danish cold buttermilk soup) analogue by fermenting tiger nut extract with plant-isolated LAB and conventional yoghurt strains. The results indicated that fermentation by *Leu. mesenteroides* (isolated from gooseberries) mimicked the acidity of commercial koldskål and generated an aroma profile similar to sweet fermented milk. Xanthan gum was, however, needed to improve the body and stability of the analogue, as fermentation alone could not reproduce the viscosity of traditional koldskål [63].

In contrast, there are numerous plant-based analogues of fermented cream products on the market (Table 3), consisting of butters, sour creams, and sour cream-based dips. Cashew, almond, coconut, and oat PBAEs often serve as the base ingredient, along with plant-based oils. Starches, gums, and plant-derived lecithin are common features in the ingredient list, likely as textural aids. Most products did not disclose the specific cultures used for fermentation. Of note, wildbrine indicates the use of lactobacilli in its butter alternatives, while Forager Project cultures its sour cream analogue with LAB that are commonly employed in yoghurt fermentation (Table 3).

#### 4.2.2. Yoghurt

Yoghurt is arguably the most popular and diverse fermented dairy analogue. Set, stirred, drinkable, flavoured—the seemingly endless variations of yoghurt products on the market belie the humble makeup of its microbiota. Traditionally, yoghurt is fermented from whole cow’s milk with only two cultures—*St. thermophilus* and *L. bulgaricus.* Though yoghurt may now be made with additional cultures such as *Lactobacillus acidophilus*, *L. casei*, and *Bifidobacterium* spp., its pool of starter cultures is comparatively small compared to cheeses and kefirs [127,136,138]. During yoghurt fermentation, lactose is fermented to form lactic acid, which gives yoghurt its signature tangy flavour and induces the acid gelation of casein, a major protein in milk. This forms the firm, viscous, and cohesive gel characteristic of dairy yoghurts [22,127]. The absence of casein in plant matrices as well as dairy-incompatible, native plant flavours are thus key hurdles towards the development of a stable, palatable, and dairy-like yoghurt analogue [22,158,161].

Commercial dairy yoghurt cultures and probiotics are typically applied in the fermentation of plant-based yoghurt analogues (Table 3 and Table 4). They have been shown to induce the formation of yoghurt-like gels, impart sweet, creamy aromas reminiscent of cow’s milk yoghurt, and in some situations, mask or reduce the perception of off-flavours such as beany notes [27,82,114,150,151,154,155]. Meanwhile, studies on suspensions of lupin protein isolate [158] and a rice–chickpea–lentil mixture [40] indicated that several novel, non-yoghurt strains are also capable of producing a yoghurt-like odour and/or texture. Like Madsen et al., these studies demonstrated that the optimal fermentation protocols for dairy analogue production may not include traditional dairy starters, which highlighted the importance of tailoring fermentation cultures and conditions to each plant matrix. This was observed by Luana et al. as well, where fermentation with *L. plantarum* LP09 (a non-dairy strain) reduced the perception of earthy, cereal notes while boosting the intensity of acid and dairy-like notes in a fermented, oat-based yoghurt drink [53]. Similarly, soy PBAE fermented with bifidobacteria produced higher levels of acetaldehyde, an important aroma compound of dairy yoghurt, compared to conventional yoghurt starters [33].

Plant-based yoghurts produce weaker gels than their dairy counterparts and are highly prone to syneresis, regardless of the type of raw material used [36,57,153,156]. While the discussed processing strategies (Section 3) are helpful, researchers have been developing fermentation protocols which can simultaneously achieve optimal flavour and texture in the final yoghurt analogue. This has led to the use of EPS-producing LAB, which synthesise and excrete largely taste-neutral EPSs that exhibit hydrocolloidal behaviour and bind water efficiently, improving the growth medium’s rheological and textural properties [26,48,49,158]. EPS-producing LAB are often isolated from yoghurt, kefir, and sourdough starters, though some are associated with beer spoilage. Beyond serving as a source of microbial-derived EPSs for food applications, EPS-producing LAB have been used in fermentations to improve the texture of a wide range of products [162,163].

Fermentation with EPS-producers has been explored in plant-based yoghurt analogues made from soy PBAE, lupin protein isolate, and quinoa flour [26,48,49,158]. Li et al. compared the effects of soy yoghurt fermentation using EPS-producing LAB versus a commercial yoghurt starter culture. As well as producing the highest apparent viscosity, the content of beany flavour compounds (hexanal, 2-pentylfuran, 2-pentanone) decreased in soy yoghurt after fermentation with an EPS-producing *L. plantarum* strain, while 3-hydroxy-2-butanone, a characteristic flavour compound of fermented dairy milk, increased to detectable levels [26]. Similarly, high viscosity was observed in the fermentation of quinoa-based yoghurt with EPS-producing *Weissella cibaria* and *W. confusa*, the latter of which also imparted sweet and dairy-like acidity [48,49]. A survey of 30 different LAB strains in yoghurts made from lupin protein isolates—which possess weak gelling ability—revealed that EPS-producers (*L. plantarum*, *Pe. pentosaceus*, *L. brevis*) were the most effective in emulating both the aroma and texture of dairy yoghurt [49,158].

Fermented, plant-based yoghurt analogues on the market are almost exclusively made from nuts, drupes, and seeds (cashew, almond, coconut). Save for Alpro’s oat yoghurts, which feature the cheese starters *Lc. lactis* and *Lc. cremoris*, most brands use traditional yoghurt cultures and their associated probiotic lactobacilli (Table 3). Starch, gums, and/or pectins appear in every ingredient list. EPS-producing LAB do not appear to be used for commercially available yoghurt analogues; if they are, they remain unspecified. A variety of starter cultures are also available for at-home fermentation of plant-based yoghurts; most include dairy yoghurt starter cultures and probiotics (Table 3).

#### 4.2.3. Cheese

Variations in milk source, starter and adjunct cultures, fermentation conditions, ripening operations, and other cheesemaking processes are responsible for the close to 1500 cheese varieties worldwide [13,137,138]. Despite significant differences in flavour, texture, and appearance among cheese varieties, cheesemaking generally results in a common aim—the production of a viscoelastic curd as a result of the agglomeration of caseins in fluid milk [13,164]. Starter cultures in cheese, namely LAB, acidify the matrix via lactose fermentation to promote cheese curd formation. They can be mesophilic (*Lc. lactis* and *Lc. cremoris*) or thermophilic (*St. thermophilus*) in nature. Adjunct cultures flourish in the cheese environment during the later ripening stage, where they metabolise various substrates to produce gas, colour, or characteristic flavour compounds in cheese [137,164].

Due to their larger molecular size and substantial structural differences, plant proteins do not exhibit similar aggregation or gelation behaviours as casein micelles [10,11]. While the inclusion of fillers and stabilisers are helpful, recent studies have highlighted the significant influence of extraction and processing parameters on the texture of cheese analogues (Table 2) [18,159,165]. Though fermentation and its resultant acidification have been used to curdle PBAEs [29,81,160], it is rarely explored as a specific strategy for textural improvement. Fermentation by LAB and commercial cheese cultures was, however, observed to improve gel hardness and reduce syneresis in soy-based petit-suisse (fresh cheese) analogues [29] and pea protein isolate–olive oil emulsions [41]. It is important to note that product formulation, including the use of gums and vegetable fats, played a significant role in the textural stability of the aforementioned gels.

Other researchers have turned to the use of fungi instead, particularly *G. candidum*, an adjunct culture responsible for the velvety appearance of Camembert and Reblochon, among other cheeses. As well as raising the pH of the cheese matrix via lactate metabolism, *G. candidum* is prized for its strong proteolytic and lipolytic activity and its ability to produce volatile sulphur compounds (VSCs) [137]. Łopusiewicz et al. [152] studied the production of a Camembert analogue by fermenting flaxseed oil cake with LAB starters (*Lc. lactis*, *Lc. Cremoris*, and *St. thermophilus*), *P. camemberti*, and/or *G. candidum.* The inclusion of *G. candidum* resulted in a significantly lower hardness and chewiness in the Camembert analogue, which is desired in soft cheeses [137]. In another study on soy-based soft cheese analogues [28], the inclusion of *G. candidum* produced a softer, stickier texture compared to pure LAB starters (*L. bulgaricus*, *St. thermophilus*), which was attributed to the high degree of protein and fat degradation observed in the former samples. In this respect, cultures with strong enzymatic activities, especially proteolytic and lipolytic pathways, could be evaluated for the textural improvement of plant-based cheese analogues. EPS-producing LAB are potential candidates as well, and some strains have already been shown to improve structure in fat-reduced dairy cheeses [166,167].

Reproducing the authentic flavour of dairy-based cheeses is its own unique challenge. Proteins and fats in plant-based ingredients differ significantly from those found in animal milk [9,10,18]; naturally, most are perceived as off-flavours in the context of dairy cheeses. Ben-Harb et al. reported a reduction in green aldehydes during pea gel fermentation with cheese-isolated cultures [159]. They also observed that the overall volatile profiles differed substantially among gels made from pea PBAE, cow’s milk, or a mixture of both, even when identical cultures were used, emphasizing the fact that cultures may display different fermentation characteristics in different matrices. Beyond this, none of the studies within the scope of our review specifically evaluated the reduction in off-flavours and/or the evolution of cheese and dairy-like notes in plant-based cheese analogues. Instead, sensory evaluation was performed to assess product likeability or to rate textural attributes; dairy benchmarks were rarely included [28,32,81].

Many fermented, plant-based cheese analogues on the market are made from cashews and almonds (Table 3). Unlike yoghurt analogues, a wide array of starter cultures is used in commercial cheese analogues, ranging from lactobacilli (direct inoculation or from yoghurt base) to water kefir and koji. Textural aids, however, are equally common in cheese analogue formulations. Interestingly, cauliflower and hemp serve as primary ingredients in a cheese sauce analogue developed by Grounded, which appears to use shio koji as its starter culture (Table 3). This deviates significantly from the overwhelming number of nut/drupe-based or occasionally grain-based cheese analogues on the market. With the exploration of non-dairy cultures and fermentation techniques, the market may soon welcome cheese and dairy analogues made from a greater variety of plant-based raw materials.

#### 4.2.4. Kefir

Originally consumed in the region of Caucasia, kefir has exploded in popularity in recent years thanks to its various health benefits. Dairy kefir is traditionally made by fermenting milk with milk kefir grains, which owes its cauliflower-like appearance to the EPS, kefiran. A symbiotic consortium of LAB, yeasts, and acetic acid bacteria (AAB) is embedded in kefiran, which confers a sour, fizzy, and mildly alcoholic taste along with a viscous texture to fermented kefir. *L. kefiranofaciens*, *L. kefiri*, *Lactococcus* spp., *Acetobacter pasteurianus*, and *Saccharomyces* spp. have been identified as the major microbiota in milk kefir grains [136]. Water kefir grains, on the other hand, are used to ferment dairy-free, fruit-based sugar solutions to create a sparkling, acidic beverage. Instead of kefiran, their EPS is composed of α-glucans, and the fermentation conditions favour the predominance of a greater variety of AAB, yeasts (*Saccharomyces* spp., *Dekkera bruxellensis*), and the LAB species *Lactobacillus nagelii* and *Lactobacillus hilgardii* [168].

Many kefir fermentation studies with both milk and water kefir grains are directed at reaping its health benefits in a plant-based matrix instead of producing something that tastes and looks like authentic dairy kefir [169]. While there have been novel studies on the production of kefir-like products from walnut and soy PBAEs [170,171], flaxseed oil cake [172], and even apple juice [173], their organoleptic properties have not been evaluated against a dairy kefir benchmark. Hence, they may not possess the dairy organoleptic qualities required of a dairy analogue (i.e., the incubation of milk kefir grains in plant matrices does not necessarily result in a dairy kefir analogue).

A recent study by Yépez et al. evaluated milk kefir and water kefir grains in the fermentation of gelatinised flour suspensions made from oat, maize, or barley against a cow’s milk control [124]. Though the study’s main purpose was to determine the feasibility of in situ riboflavin fortification via co-fermentation of LAB with kefir grains, the viscosities and volatile profiles of the grain-based kefir analogues were also analysed. Milk kefir grains were found to present a better aptitude for lactic acid production and viscosity improvement, both quality indicators of dairy kefir. In particular, *L. plantarum* M5MA1-B2 further improved acetic acid and lactic acid content in maize kefir and oat kefir, respectively, and it enhanced viscosity when co-inoculated with water kefir, which highlighted the potential of using non-milk kefir strains in improving the quality of plant-based dairy kefir analogues.

There is a comparatively smaller offering of plant-based dairy kefir analogues on the market, most of which are made from coconut PBAE and require stabilisers in the form of starch. (Table 3). These include recent product launches, which are aligned with the new and growing interest in such analogues. Commercial dairy kefir analogues may, however, face stiffer competition compared to other dairy product analogues. As well as contending with their dairy counterparts, dairy kefir analogues also face existing competition in the plant-based sector in the form of water kefirs. Thus, organoleptic improvement is even more critical to engage and sustain consumer interest with a nutritious, tasty, and visually appealing product.

#### 4.2.5. Targeted Fermentation and Precision Fermentation

Instead of the traditional fermentation of raw materials to create a plant-based dairy analogue, some researchers have used highly targeted fermentation approaches to eliminate or produce specific flavour compounds in plant-based raw materials, which may be further processed into dairy analogues or extracted to obtain flavour compounds of interest. For example, fungal fermentation of soy PBAE with *Agrocybe aegerita* successfully produced short chain fatty acids (SCFAs) reminiscent of Parmesan and Emmental cheese [174], while LAB and/or yeast fermentation of pea protein isolate was shown to reduce its green, leguminous, and bitter taste attributes, which are dairy-incompatible flavours [175,176,177]. In particular, Garcia Arteaga et al. reported the evolution of cheesy and salty notes after fermentation, though protein functionality was negatively affected [177]. Diacetyl and acetaldehyde, both important compounds in dairy-like aroma, are frequently reported in LAB fermentation of cereal- and soy-based matrices [33,65,178].

Precision fermentation processes have also been employed with genetically modified microorganisms (bacteria, yeast, or fungi) to synthesise dairy proteins and fats, which are then used as a base to create conventional dairy products. The reader is invited to refer to a recent review by Mendly-Zambo et al. [3] on this topic, which covers start-ups such as Perfect Day and the Real Vegan Cheese project [3]. Similar disruptors in this sphere include Change Foods, New Culture, Legendairy Foods, Better Dairy, and Remilk. The use of genetic modification (GM) technology, however, has always faced significant consumer resistance. Coupled with the relatively high cost of microbial engineering, much investment and research are expected before GM-produced dairy becomes commercially competitive against conventional dairy products or plant-based dairy analogues [3].

## 5. Conclusions

Plant-based dairy analogues represent a rapidly growing market segment, and the food industry has responded to consumer demand by developing a wide range of such dairy analogues from yoghurt to cheese, covering a range of plant materials from almonds to potatoes. The success of these products will greatly hinge on their organoleptic qualities in terms of aroma, taste, and texture, as well as other features such as stability and nutritional properties. The relative novelty of these products has resulted in the application of fermentation and other innovative processing methods on top of the exploration of a wide range of raw materials, consequently illustrating the vast options for scientists, technologists, and the industry. Fermentation has shown promising results in terms of imparting flavour and/or texture reminiscent of conventional dairy, especially in applications related to fermented dairy products such as yoghurt and cheese. The use of microorganisms not typically associated with dairy environments, e.g., EPS-producers, is also an avenue worth further exploration. Nonetheless, care needs to be taken to select and design products and processes that can match or surpass the organoleptic qualities of conventional bovine milk products so that they remain competitive in the market. Consequently, the application of technologies different from those used in the traditional dairy industry are expected since plant materials possess different physicochemical qualities and greater heterogeneity. It is anticipated that plant-based dairy analogues will continue to surge in popularity, and while organoleptic properties will remain the top factor in increasing consumer acceptability, greater efforts are warranted in making such products nutritionally whole and sustainable to produce, both for the wellbeing of consumers and the environment.

## Figures and Tables

**Table 1:** Carbohydrate, fibre, total protein, and total fat content of some common plant-based raw materials (all values are expressed in g/100 g dry basis).

		Carbohydrate	Fibre	Protein	Fat
Legumes	Soy [4]	30.2	9.3	36.5	19.9
	Chickpea [4]	63.0	12.2	20.5	6.0
	Lupin [4]	40.4	18.9	36.2	9.7
	Faba bean [4]	58.3	25.0	26.1	1.5
	Lentil [4]	63.1	10.8	23.9	2.2
	Peanut [19]	16.1	8.5	25.8	49.2
Grains	Rice [20]	80.0	1.3	7.1	0.7
	Oat [20]	66.3	11.6	16.9	6.9
	Quinoa [20]	64.2	14.2	14.5	5.2
	Maize [20]	74.0	7.3	9.4	4.7
	Barley [20]	77.7	15.6	9.9	1.2
Nuts, Drupes, and Seeds	Almond [19]	51.6	12.5	21.2	49.9
	Hazelnut [19]	16.7	9.7	15.0	60.8
	Cashew [19]	30.2	3.3	18.2	43.9
	Walnut [19]	13.7	6.7	15.2	65.2
	Macadamia [19]	13.8	8.6	7.9	75.8
Tubers	Potato [21]	15.7	2.4	1.7	0.1
	Cassava [21]	38.1	1.8	1.4	0.3
	Yam [21]	27.9	4.1	1.5	0.2
	Sweet potato [21]	20.1	3.0	1.6	0.1

Values are obtained from the following literature: Cichonska and Ziarno (2022) [4]; Souza et al. (2015) [19]; Petrova and Petrov (2020) [20]; Chandrasekara and Kumar (2016) [21].

**Table 2:** Applications of various processing techniques for the extraction of plant-based ingredients intended for dairy analogue production.

Technique	Source	Material	Process Application	Changes in Functional/ Textural Properties	Influence on Flavour/ Off-Flavour Formation	Impact on Nutrients/ Antinutrients
Mechanical Pre-Treatment and Extraction						
Roasting	Ferawati et al. (2019) * [67]	Pulses (yellow pea, gray pea, faba bean, white bean)	Roasting before flour production	Increase in water-holding capacity (WHC)	-	Increase in total dietary fibre; Some pulses experienced increase in choline and folate
	Zaaboul et al. (2019) * [69]	Peanuts	Roasting before aqueous extraction	Higher protein solubility and extraction; Improved emulsion stability	-	-
	Ahmadian-Kouchaksaraei et al. (2014) * [70]	Sesame	Roasting before aqueous extraction	-	Reduced LOX activity; Reduced bitterness, ‘beany’ and ‘chalky’ flavours; Reduced oxidative off-flavour formation	-
Dehulling	Ma et al. (2021) [71]	Peas	Dehulling before aqueous extraction (yoghurt fermentation)	-	Reduced formation of the off-odorant 2-methoxy-3-isopropyl-(5/6)-methylpyrazine	Lowered amounts of extracted albumin (no effect on texture)
	Ghavidel andPrakash (2007) * [72]	Legumes (green gram, cowpea, lentil, chickpea)	Dehulling before flour production	-	-	Decreased soluble and insoluble dietary fibre; Decreased phytate; Increased iron and calcium bioavailability
Soaking and blanching	Ma et al. (2021) [71]	Peas	Blanching before aqueous extraction (yoghurt fermentation)	Increased firmness, viscosity and WHC of yoghurts	Reduced LOX activity and related lipid oxidation off-flavours	-
	Peng et al. (2015) [73]	Soy	Blanching before aqueous extraction (yoghurt fermentation)	Decreased soy protein solubility; Higher temperature blanching led to formation of softer, less firm yoghurts	Reduced ‘beany’ off-flavour and ‘chalky’ taste	-
	Ferawati et al. (2019) * [67]	Pulses (yellow pea, gray pea, faba bean, white bean)	Blanching/boiling before flour production	Improved WHC and gelation rates	-	Choline losses in some blanched pulse flours
Milling	Kaharso et al. (2021) * [74]	Soy	Anaerobic wet-milling for aqueous extraction	-	Reduced lipid oxidation products and off-odorants (e.g., alcohols and aldehydes)	-
	Kizzie-Hayford et al. (2015) * [64]	Tiger nut	Wet-milling with a pneumatic press	Increased milling intensity produced a smaller particle size distribution and higher total solids yield (improved PBAE colloidal stability)	-	-
Chemical and biological treatments						
pH alterations	Ma et al. (2021) [71]	Peas	Alteration of soaking pH before aqueous extraction and yoghurt fermentation	Alkaline treatment reduced gel hardness (improved sensory scores for texture)	Alkaline and acid treatments reduced lipid oxidation products and improved sensory scores for smell and taste	-
	Pineli et al. (2015) * [75]	Quinoa	Alteration of cooking pH	Optimised pH and salinity resulted in three times greater soluble protein extraction versus pure water	-	-
	Ahmadian-Kouchaksaraei et al. (2014) * [70]	Sesame	Alkalinisation of soaking water before aqueous extraction	Increased protein solubility and fat extraction versus untreated soaking water	Lower LOX activity and theoretical reduction in off-flavour formation under alkaline conditions	-
Chemical deodorisation	Vatansever et al. (2021) * [76]	Pea	Treatment with supercritical CO_2_ and ethanol extraction	-	Reduction in total volatiles and odour-contributing compounds to below detection threshold levels	-
	Guldiken et al. (2021) * [77]	Lentil	Treatment with polymeric adsorbents	-	Reduced aldehydes (potential off-odorants)	-
	Wang et al. (2020) * [78]	Pea	Washing of flour with organic solvents	Reduction in emulsion stability and solubility of pea protein flour	Removed majority of volatile compounds, resulting in deodorised product	-
	Inouye et al. (2002) * [31]	Soy	Treatment with polystyrene and zeolite-based adsorbents	-	Reduced off-odorant (hexanal) in deodorised product	-
Enzymatic treatments	Jiang et al. (2020) [79]	Faba bean	Starch hydrolysis (Termamyl^®^ Ultra 300 L) of slurry before yoghurt fermentation	Addition of enzymatic starch hydrolysate produced yoghurts with higher viscosities and gel strengths	-	-
	Zannini et al. (2018) [49]	Quinoa	Protease (Profix 100 L and Bioprotease PF50) treatment of slurry before yoghurt fermentation	Improved protein solubility in produced PBAE	-	-
	Park and Lee (2015) [80]	Soy	Flavourzyme^®^ and Neutrase^®^ treatment before yoghurt fermentation	Reduced yoghurt viscosity; Reduced WHC	Increased organic acid production during fermentation	-
	Luana et al. (2014) [53]	Oat	Enzymatic (Depol 740 L and Grindamyl 1000) treatment and yoghurt fermentation	Lower viscosity and WHC	Increased sweet and cereal taste	Significant increase in soluble fibre content
	Li et al. (2013) [81]	Soy	Enzymatic (papain) treatment of fermented or acidified coagulated PBAE for cheese production	Extensive hydrolysis of soy proteins, reducing graininess; Better stability, cheese homogeneity, and sensory acceptance	-	-
Germination	Ogundipe et al. (2021) [82]	Tiger nut	Germination before aqueous extraction and yoghurt fermentation	Reduced fat content	Decreased aroma sensory score (negative impact)	Decreased anti nutrient content (oxalate, saponin, phytate, and trypsin inhibitor)
	Cáceres et al. (2019) [83]	Rice	Germination before preparation of flour-based yoghurts	Lowered yoghurt consistency after starch hydrolysis during germination	Improved sensory acceptance after fermentation versus non-germinated rice flour; Some increase in bitterness observed (likely lipid oxidation products generated during germination)	Increased antioxidant activity and γ-aminobutyric acid content
	Hwang et al. (2018) [84]	Soy	Germination before yoghurt production	-	-	Increased *γ*-aminobutyric acid, total phenolics, and isoflavone aglycone contents
	Yang et al. (2010) [85]	Soy	Germination (various hypocotyl lengths) for the preparation of yoghurts	Decreased yoghurt WHC with increased hypocotyl length; Decreased hardness, adhesiveness, and gumminess (more sensorially acceptable yoghurt texture)	Reduced the ‘beany’ off-flavour, likely due to reduced LOX activity; Longer hypocotyl length associated with an unpleasant soybean sprout and astringent flavour; Enhanced free amino acid content may lead to pleasant flavour development during fermentation	-
Germination	Le et al. (2021) * [86]	Soy	Germination before PBAE production	Protein denaturation led to larger droplet size and lower viscosity	Increase in overall sensory acceptability, likely due to the reduction in off-flavours	Increased *γ*-aminobutyric acid content
	Lopes et al. (2020) * [24]	Pulses (Sweet lupin, chickpea, green pea, yellow pea)	Germination before PBAE production	Reduced gelation in pulse beverages due to starch hydrolysis	-	-
Other novel treatments						
High pressure homogenisation/Microfluidisation	Levy et al. (2022) [87]	Potato	HPH emulsions were fermented into yoghurts	Improved gelation and lowered creaming velocities for finer, more stable emulsions	-	-
	Demirkesen et al. (2018) [88]	Hazelnut	Microfluidisation of slurry before yoghurt fermentation	Improved WHC and higher slurry consistency (firmer yoghurts more similar to dairy yoghurt)	-	Successful production of high-fibre hazelnut yoghurt without residue removal during PBAE production
	Ferragut et al. (2009) [89]	Soy	UHPH PBAE was fermented into a yoghurt	Improved WHC, rigidity and firmness with increase in homogenisation pressure	-	-
	Xia et al. (2019) * [90]	Sweet lupin	Slurry was homogenised under high pressure to yield a PBAE	Decreased particle size, sedimentation and improved emulsion stability; Reduced viscosity	-	-
High pressure homogenisation/Microfluidisation	Jeske et al. (2019) * [91]	Lentil	Slurry was homogenised under high pressure to yield a PBAE	Reduced particle size, which increased solubility and reduced aggregation (improved stability); End product texturally comparable with other commercial PBAEs	-	-
Ultrasonication	Mu et al. (2022) * [92]	Soy	Ultrasonication of PBAE	Reduced particle size, improved thermal and emulsion stability	Decreased lipid oxidation off-odorants, including ‘grease-oxidative’ and ‘beany’ flavours	-
	Lu et al. (2019) * [93]	Coconut, maize	Ultrasonication of PBAE with maize additives	Reduced particle size, improved emulsion stability and homogenised mixture	-	-
	Abdullah et al. (2018) * [94]	Coconut	Ultrasonication of PBAE	Reduced particle size and creaming index, improving stability	-	-
High hydrostatic pressure (HHP)	Wang et al. (2021) [95]	Soy	Optimised HHP processing before yoghurt fermentation	Enhanced WHC and protein solubility Reduced yoghurt syneresis	Reduced LOX activity and related off-odour compounds	-
High hydrostatic pressure (HHP)	Sim et al. (2021) [96]	Legumes (mung bean, chickpea, pea, lentil, faba bean)	HHP processing to achieve yoghurt textures (no fermentation)	Formed pressure-induced, protein-based gels with viscoelastic properties similar to dairy yoghurt	-	-
	Dhakal et al. (2014) * [97]	Almond	HHP processing of PBAE	-	Allowed for protein modification without the formation of undesirable cooked flavours	Decreased amaldin content and hence allergenicity
Pulsed electric field (PEF)	Manzoor et al. (2020) * [98]	Almond	Comparison versus thermal treatment on PBAE	Increased colloidal stability and reduced sedimentation	Decreased LOX and peroxidase (POD) activity may result in reduced off-odour formation	Increased free amino acid content
	Li et al. (2013) * [99]	Soy	PEF treatment of PBAE	Reduction in viscosity	Decreased LOX activity may result in reduced off-odour formation	-

* Indicates that study investigated the unfermented plant ingredient (e.g., flours, PBAEs). HPH: high pressure homogenisation; UHPH: ultra-high pressure homogenisation; HHP: high hydrostatic pressure; PEF: pulsed electric field.

**Table 3:** Some examples of fermented plant-based dairy analogues on the market and commercial starter cultures.

Brand	Product	Ingredients/Application	Cultures (If Specified) ^1^
Fermented cream products			
Forager Project	Organic Dairy-Free Sour Cream	Coconut and cashew milk (filtered water, coconut cream, cashews), tapioca starch, sea salt, pectin, distilled vinegar, lactic acid, locust bean gum, tricalcium phosphate, agar, live active cultures	*St*. *thermophilus*^2^, *L. bulgaricus*, *L. acidophilus*, *B. bifidus*, *Lc. lactis*, *L. plantarum*
Good Karma	Plant-Based Sour Cream Dairy-Free Alternative	Water, coconut oil, tapioca flour, pea protein, dextrose, corn starch (unmodified, identity preserved), tricalcium phosphate, sea salt, sunflower lecithin, lactic acid (vegan), natural flavour, vitamin A palmitate, vitamin D2, vitamin B12, live and active cultures	Unspecified
Kite Hill	Sour Cream Alternative	Almond milk (water, almonds), coconut oil, rice starch, coconut milk, maltodextrin, chickpea protein, salt, cultures	Unspecified
	Tzatziki	Almond milk (water, almonds), cucumbers, rice starch, tapioca flour, salt, garlic, dill, onions, maltodextrin, lemon juice concentrate, locust bean gum, black pepper, natural flavour, live active cultures	Unspecified
	European Style Butter Alternative	High oleic sunflower oil, cultured almond milk (water, almonds, cultures), coconut oil, natural flavours, cocoa butter, cultured dextrose, sea salt, sunflower lecithin, lactic acid, *β*-carotene (for colour)	Unspecified
Lauds	Cultured Oat Butter	Organic coconut oil, oat milk yoghurt (water, organic oats, yellow split peas, potato starch, natural cultures), sunflower oil, non-GMO soy lecithin, Tasmanian sea salt, natural colour (*β*-carotene), preservative (sorbic acid)	Unspecified (yoghurt base)
Miyoko’s Creamery	European Style Cultured Vegan Butter (Unsalted)	Organic coconut oil, organic cultured cashew milk (filtered water, organic cashews, cultures), filtered water, organic sunflower oil, contains 2% or less of: organic sunflower lecithin, organic cultured dextrose, natural flavours derived from oregano, flaxseed, and plums, lactic acid	Unspecified
	Spreadable Cultured Vegan Oat Milk Butter	Organic sunflower oil, organic cultured whole grain oat milk (filtered water, cultured organic oats), organic coconut oil, contains less than 2% of organic sunflower lecithin, sea salt, organic cultured dextrose, lactic acid, natural flavours derived from oregano, flaxseed, and plums	Unspecified
The Vegan Dairy	Cultured Butter	Organic cashew nuts, organic coconut oil, cold pressed rice bran oil, filtered water, natural vegan cultures, sea salt, soy lecithin, natural vegan colouring derived from sunflowers	Unspecified
wildbrine	wildCREAMERY Sour Cream Alternative	Water, sunflower oil, coconut oil, cashews, tapioca flour; contains 2% or less of: oats, chickpeas, sea salt, cabbage, cultured dextrose	Lactobacillus cultures
	wildCREAMERY Oat Butter Alternative	Coconut oil, sunflower oil, oat milk (water, oats), water, potato, sweet potato; contains 2% or less of: sunflower lecithin, sea salt, cabbage, culture dextrose	Lactobacillus cultures
	wildCREAMERY European Style ButterAlternative	Coconut oil, water, sunflower oil, cashews; contains 2% or less of: sunflower lecithin, yam, sea salt, cabbage, oats, cultured dextrose	Lactobacillus cultures
Yoghurt and drinkable yoghurt			
Alpro	Plain No Sugars	Soya base (water, hulled soya beans (10.7%)), calcium (tricalcium citrate), acidity regulators (sodium citrates, citric acid), stabiliser (pectins), natural flavouring, sea salt, antioxidants (tocopherol-rich extract, fatty acid esters of ascorbic acid), vitamins (B12, D2), yogurt cultures	*St. thermophilus*, *L*. *bulgaricus*
	Greek Style Plain	Soya base (water, hulled soya beans (15.7%)), sugar, stabiliser (pectins), calcium (tricalcium citrate), acidity regulators (sodium citrates, citric acid), natural flavouring, sea salt, antioxidants (tocopherol-rich extract, fatty acid esters of ascorbic acid), vitamins (B12, D2), yogurt cultures	*St. thermophilus*, *L. bulgaricus*
	Greek Style Coconut	Coconut milk (45%) (coconut cream, water), water, coconut water (20%), modified starch, thickeners (pectin, agar), natural flavourings, sea salt, cultures	*St. thermophilus*, *L bulgaricus*, *B. lactis*
	Greek Style Oat	Oat base (water, oat (11.1%)), sunflower oil, modified starches, soluble corn fibre, pea protein, calcium (tricalcium phosphate), acidity regulators (malic acid, citric acid, sodium citrates), thickeners (pectin, agar), natural flavourings, cultures, vitamins (B12, D2)	*St. thermophilus*, *Lc. cremoris*, *Lc. lactis*, *L. casei*, *L. acidophilus*
	Absolutely Oat	Oat base (water, oat (15.5%)), modified starch, chicory root fibre, pea protein, sunflower oil, natural flavouring, cultures	*Lc. lactis*, *B. lactis*, *L. acidophilus*, *St. thermophilus*, *L. bulgaricus*
Cocobella	Dairy-Free Coconut Yoghurt	Coconut yoghurt (water, coconut milk, native starch, tapioca syrup, carob bean extract, agar, yoghurt cultures and probiotics)	*Bifidobacterium* spp., *L. acidophilus*, *L. bulgaricus*, *L. paracasei*, *St. thermophilus*
Cocos Organic	Organic Coconut Milk Yoghurt Alternative	Organic coconut milk (98%), organic tapioca starch, live vegan cultures	Unspecified
COYO	Natural Organic Coconut Milk Yoghurt Alternative	Organic coconut milk (97%), organic tapioca starch, live vegan cultures	Unspecified
Forager Project	Cashew milk Dairy-Free Yogurt	Cashew milk (filtered water, cashews), tapioca starch, locust bean gum, coconut cream, live active cultures	*St. thermophilus*, *L. bulgaricus*, *L. acidophilus*, *B. bifidus*, *Lc. lactis*, *L. plantarum*
	Unsweetened Drinkable Yogurt	Cashew milk (filtered water, cashews), tapioca starch, coconut cream, live active cultures	*St. thermophilus*, *L*. *bulgaricus*, *B. bifidum*, *B. lactis*, *L. acidophilus*, *L. casei*, *L. paracasei*, *L. plantarum*, *L. rhamnosus*
Kite Hill	Almond Milk Yogurt (Plain)	Almond milk (water, almonds), cane sugar, starch, citrus fiber, locust bean gum, xanthan gum, live active cultures	*St. thermophilus*, *L. bulgaricus*, *L. acidophilus*, bifidobacteria
	Blissful Coconut Milk Yogurts (Plain Unsweetened)	Coconut cream (water, coconut), modified tapioca starch, salt, live active cultures, vitamin D2, vitamin B12	*St. thermophilus*, *L. bulgaricus*, *L. acidophilus*, bifidobacteria
	Greek Style Yogurts (Plain Unsweetened)	Almond milk (water, almonds), soy protein isolate, tapioca starch, natural flavours, live active cultures	*St. thermophilus*, *L. bulgaricus*, *L. acidophilus*, bifidobacteria, *L. plantarum*
Nush	Dairy-Free Organic Almond Yog (Natural)	Organic almond milk (95%) (filtered water/organic almonds), organic tapioca starch, thickener: organic carob gum, live vegan cultures	Unspecified
Oatly	Oatgurt Plain	Oat milk (water, oats), low erucic acid rapeseed oil, potato starch. Contains 2% or less of: dextrose, pea protein, potato protein, calcium carbonate, guar gum, tricalcium phosphate, locust bean gum, live active cultures)	*St. thermophilus*, *L. bulgaricus*, *L. casei*, *L. acidophilus*, *B. lactis*
Raglan Food	Dairy-Free Coconut Yoghurt (Natural Greek Style)	Organic coconut cream, natural starch (corn), live vegan cultures	*L. acidophilus*, *Bifidobacterium* spp.
Sojade	Greek Style Soya Yogurt Alternative, Organic	Soya drink 99% (water, soya beans 12.5%), thickener: pectin, selected ferments	*L. acidophilus*, *Bifidobacterium* spp.
Cheese			
Forager Project	Vegan Jack	Forager Project Yogurt (cashew milk (filtered water, cashews), tapioca starch, coconut cream, cultures), water, coconut oil, maize starch, tapioca starch, sea salt, calcium phosphate, natural flavours, fava bean protein flour, cultured dextrose, lactic acid	Unspecified
Grounded	Cheese Free Cheese Sauce	Filtered water, cauliflower, coconut oil, sunflower oil, shio koji, gluten-free oats, rice starch, hemp seed, less than 2% of: tapioca starch, sea salt, spices, citrus fiber, sodium citrate, lactic acid, yeast extract, organic agave nectar, guar gum, mushroom powder, onion powder, *β-*carotene	Shio koji (likely)
Kite Hill	Garlic and Herb Soft Spreadable Cheese	Almond milk (water, almonds), dehydrated garlic, dehydrated onions, salt, rice starch, cane sugar, spices, potato starch, mushroom extract, enzyme, tartaric acid, cultures	Unspecified
	Almond Milk Ricotta Alternative	Almond milk (water, almonds), salt, enzymes, tartaric acid, cultures	Unspecified
	Cream Cheese Alternative (Plain)	Almond milk (water, almonds), salt, enzyme, xanthan gum, guar gum, mushroom extract (to help preserve freshness), lactic acid, citric acid, cultures	Unspecified
Lauds	Original Oat Melt	Oat milk yoghurt (water, organic oats, yellow split peas, potato starch, natural cultures), water, tapioca flour, sunflower oil, cashews, nutritional yeast, organic coconut oil, Tasmanian sea salt, carrageenan, miso paste (from soy), spices, olive oil, natural colour (*β-*carotene), preservative (sorbic acid)	Unspecified (yoghurt base)
	Aged Cashew Cheese	Cashews, organic coconut oil, water kefir (water, natural cultures, sugar), miso paste (from soy), nutritional yeast, spices, Tasmanian sea salt, preservative (sorbic acid)	Water kefir
	Ashed Walnut Cheese	Cashews, Tasmanian walnuts, coconut oil, water kefir (water, natural cultures, sugar), miso paste (from soy), nutritional yeast, spices, Tasmanian sea salt, activated charcoal, preservative (sorbic acid)	Water kefir
	Almond Persian Feta	Almonds, organic coconut oil, water, sunflower oil, oat milk yoghurt (water, organic oats, yellow split peas, potato starch, natural cultures), Tasmanian sea salt, preservative (sorbic acid)	Unspecified (yoghurt base)
Miyoko’s Creamery	Organic Cultured Vegan Cream Cheese (Classic Plain)	Organic cashews, filtered water, organic coconut cream, sea salt, cultures	Unspecified
	Organic Cashew Milk Mozzarella	Organic cashew milk (filtered water, organic cashews), organic coconut oil, organic tapioca starch, sea salt, organic agar, mushroom extract, organic konjac, cultures	Unspecified
Miyoko’s Creamery	Liquid Vegan Pizza Mozzarella	Plant milk (filtered water, organic cashews), organic sunflower oil, organic tapioca starch, sea salt, mushroom extract, organic sunflower lecithin, organic konjac, cultures	Unspecified
	Aged Sharp English Farmhouse Cashew Milk Cheese	Organic cashew milk (organic cashews, filtered water), organic chickpea miso (organic rice koji (organic rice, koji spores), organic whole chickpeas, sea salt, water), nutritional yeast, sea salt, natural flavours (derived from oregano, plum, flaxseed), cultures	Chickpea miso (rice koji), unspecified
Nush	Creamy Almond M·lk Spread (Plain)	Almond milk (95%) (filtered water/almond), potato starch, thickener: carob gum, salt, thickener: transglutaminase, live vegan cheese cultures	Unspecified
Nut Culture	Badass Pepper Jack— Plant-Based Cheese Wheel	Organic cashew nuts, deactivated yeast, jalapeño flakes, chili flakes, salt, vegan cultures	Probiotic cultures (unspecified)
plant perks	Sriracha Cheddar Plant-Based Cheeze Spread	Cashews, filtered water, sriracha (jalapeño peppers, water, sugar, distilled vinegar, salt, garlic powder, xanthan gum), MCT oil, sea salt, onion powder, garlic powder, nutritional yeast, cultures	Unspecified
Silk^®^	Plain Almond milk Dairy-Free Yogurt Alternative	Almond milk (filtered water, almonds), cane sugar, pectin, calcium citrate, calcium phosphate, vitamin D2, live and active cultures	Unspecified
	Plain Soy milk Dairy-Free Yogurt Alternative	Soymilk (filtered water, soybeans), cane sugar, corn starch, tricalcium phosphate, pectin, natural flavour, dipotassium phosphate, sea salt, citric acid, live and active cultures, mixed tocopherols and vitamin C ester (to protect freshness), vitamin D2	Unspecified
	Vanilla Greek Style Coconut milk Yogurt Alternative	Coconut milk (filtered water, coconut cream), water, pea Protein, cane sugar, natural flavours, pectin, calcium phosphate, cinnamon, salt, live and active cultures, vitamin D2.	Unspecified
The Vegan Dairy	Dill Chèvre	Organic cashew nuts, organic coconut oil, filtered water, sea salt, natural vegan cultures, dill leaves	Unspecified
wildbrine	wildCREAMERY Cream Cheese Alternative	Water, coconut oil, sunflower oil, cashews, coconut cream, tapioca flour; contains 2% or less of: chickpeas, cabbage, oats, sea salt, sunflower lecithin, cultured dextrose	Lactobacillus cultures
	wildCREAMERY Brie Alternative	Organic cashews, water, organic coconut oil, organic coconut cream, contains 2% or less of: organic nutritional yeast, organic cabbage, organic oats, sea salt	Lactobacillus cultures, *P. candidum*
Kefir			
Biotiful	Plant-Based Oat Kefir Original	Oat base (water, gluten-free oats (11%), sunflower oil, salt), coconut cream, stabilisers (tapioca starch, pectin), rice flour, fruit extracts (apple, carob, grape), natural flavouring, lemon concentrate, vitamin B12, vitamin D2, calcium phosphate, live vegan kefir cultures	*Bifidobacterium* spp., *L. acidophilus*, *L. bulgaricus*, *L. rhamnosus*, kefir (unspecified)
Cocobella	Kefir Probiotic Yogurt	Coconut yoghurt (water, coconut milk, coconut oil, tapioca syrup, tapioca starch, carob bean extract, yoghurt cultures and probiotics)	*B. lactis*, *L. acidophilus*, *L. bulgaricus*, *L. paracasei*, *St. thermophilus*
Cocos Organic	Organic Coconut Milk Kefir	Organic coconut milk (98%) (filtered water, organic coconut), thickener: organic tapioca starch, live vegan kefir cultures	Kefir (unspecified), *B. lactis* BB12
COYO	Natural Organic Coconut Milk Kefir	Organic coconut milk (50%), filtered water, organic tapioca starch, live vegan kefir cultures	Unspecified
Raglan Food	Dairy-Free Probiotic Kefir	Organic coconut milk (water, coconut), natural starch, live vegan probiotics, vitamin C	*L. acidophilus*, *Bifidobacterium* spp., *St. thermophilus*, *L. bulgaricus*, *L. plantarum*, *L. paracasei*
Sojade	Natural Soya Kefir Alternative, Organic	Soya juice (97.4%), apple juice concentrate, live vegan kefir cultures 0.03%	Kefir (unspecified)
Commercial starter cultures			
belle and bella	Non-Dairy Yogurt Starter	Yoghurt analogue starter for various plant bases	*L. bulgaricus*, *St. thermophilus*, *L. acidophilus*
Chr Hansen	VEGA™ Culture Kit	Yoghurt analogue starter for various plant bases	Eleven cultures, unspecified, contains probiotics
Cultures for Health	Vegan Yogurt Starter Culture	Yoghurt analogue starter for various plant bases	*B. bifidum*, *L. acidophilus*, *L. casei*, *L. bulgaricus*, *L. rhamnosus*, *St. thermophilus*
DuPont™ Danisco^®^	VEGE Cultures	Dairy analogue starter for various plant bases	Unspecified, contains probiotics
Sacco System	4Choice	Dairy analogue starter for various plant bases	Unspecified
Vivo	Probiotic Vegan Yoghurt	Yoghurt analogue starter for soy base	*St. thermophilus*, *L. bulgaricus*, *L. acidophilus* (NCFM^®^), *L. paracasei* (Lpc-37), *B. lactis* (Bi-07), *B. lactis* (Bl-04), *B. bifidum*, *B. infantis*, *L. gasseri*, *L. rhamnosus*, *L. pentosus*, *L. plantarum*

^1^ Abbreviations of the microbial genus names are as follows: *Lactobacillus* (L.); *Penicillium* (P.); *Streptococcus* (St.); *Bifidobacterium* (B.); *Lactococcus* (Lc.). ^2^ The complete scientific name of each strain is always provided where possible e.g., *St. thermophilus*, *L. bulgaricus*.

**Table 4:** Selected studies in the past ten years (2012 to date) applying fermentation as a strategy to improve the organoleptic properties of plant-based dairy analogues.

Authors	Benchmark/Application	Raw Material	Fermentation Strategy	Culture(s) ^1^	Source of Culture
Milk and other milk-based products					
Tangyu et al. (2021) [37]	Cow’s milk	Chickpea	Single culture (*Bacillus* spp., LAB)	*Bac. amyloliquefaciens* NCC 156, *L*. *paracasei* ^2^ NCC 2511	Various (for *L*-lysine metabolism)
Fermented cream products					
Madsen et al. (2021) [63]	Buttermilk koldskål	Tiger nut	Single or mixed culture (LAB)	*Leu. mesenteroides* DK71, *Leu. citreum* DK93, *Pe. pentosaceus* DK103, *Lc. lactis* DK130, *L. plantarum* DK293, YFM ^3^ (*L. bulgaricus*, *St. thermophilus*)	Legumes, vegetables, fruits, dairy yoghurt
Yoghurt and drinkable yoghurt					
Khrundin et al. (2021) [150]	Milk yoghurt	Soy, oat, buckwheat	Mixed culture (LAB)	Classic yoghurt fermentation (unspecified)	Dairy yoghurt
Ogundipe et al. (2021) [82]	Milk yoghurt	Tiger nut	Mixed culture (LAB)	*L. bulgaricus*, *St. thermophilus*	Dairy yoghurt
Yang et al. (2021) [114]	Milk yoghurt	Pea, mung bean	Mixed culture (LAB)	VEGE 022 (*St. thermophilus*, *L. bulgaricus*, *L. plantarum*, *L. acidophilus* NCFM^®^, *B. lactis* HN019™) YF-L904 (*St. thermophilus*, *L. bulgaricus*)	Dairy yoghurt
Aydar et al. (2021) [151]	Milk yoghurt	Jerusalem artichoke, almond	Mixed culture (LAB)	Vivo Active (*L. bulgaricus*, *St.* *thermophilus*, *L. acidophilus*, *B. lactis*)	Dairy yoghurt
Łopusiewicz et al. (2020) [152]	Milk yoghurt	Flaxseed	Mixed culture (LAB)	VIVO-AKTIV (*L. bulgaricus*, *St.* *thermophilus*, *L. acidophilus*, *B. lactis*)	Dairy yoghurt
Pachekrepapol et al. (2020) [57]	Milk yoghurt	Coconut	Mixed culture (LAB)	YF-L812 (*L. bulgaricus*, *St. thermophilus*)	Dairy yoghurt
Pontonio et al. (2020) [40]	Milk yoghurt	Rice, chickpea, lentil	Mixed culture (LAB)	*L. plantarum* DSM33326, *L. brevis* DSM33325 *	Plant matrices
Raikos et al. (2020) [153]	Milk yoghurt	Oat	Mixed culture (LAB)	Yo-Mix^®^ ABY yogurt culture (*B. lactis*, *L. acidophilus*, *L. bulgaricus*, *St. thermophilus*, *L. lactis*)	Dairy yoghurt
Brückner-Gühmann et al. (2019) [154]	Milk yoghurt	Oat	Mixed culture (LAB)	YC-X11 Yo-Flex (*L. bulgaricus*, *St. thermophilus*)	Dairy yoghurt
Ani et al. (2018) [155]	Milk yoghurt	Soy, *Moringa oleifera* seeds, bambara groundnut	Mixed culture (LAB)	*L. bulgaricus*, *St. thermophilus*	Dairy yoghurt
Ermiş et al. (2018) [156]	Milk yoghurt	Hazelnut	Mixed culture (LAB)	*L. bulgaricus*, *St. thermophilus*	Dairy yoghurt
Lorusso et al. (2018) [48]	Milk yoghurt drink, commercial plant-based analogues	Quinoa	Single culture (LAB)	*L. rhamnosus* SP1, *W. confusa* DSM 20194, *L. plantarum* T6B10	Sourdough, probiotics, EPS-producer
Wang et al. (2018) [36]	Milk yoghurt	Soy, chickpea	Mixed culture (LAB)	belle and bella (*L. bulgaricus*, *St. thermophilus*, *L. acidophilus*)	Dairy yoghurt
Zannini et al. (2018) [49]	Milk yoghurt	Quinoa	Single culture(LAB)	*W. cibaria* MG1	Sourdough
Bansal et al. (2016) [106]	Milk yoghurt	Peanut	Single culture (LAB)	*L. brevis* MTCC no. 1750, *L. casei* MTCC no. 1423, *L. fermentum* (MTCC no. 903, MTCC no. 1745, BBE4, BBE5), *L. plantarum* (MTCC no. 6160, MTCC no. 1407), *St. faecalis* T110	Probiotics
Falade et al. (2015) [157]	Milk yoghurt	Soy, bambara groundnut	Mixed culture (LAB)	*L. bulgaricus*, *St. thermophilus*	Dairy yoghurt
Pandey and Mishra (2015) [27]	Milk yoghurt	Soy	Mixed culture (LAB)	*L. acidophilus* NCDC11, *St. thermophilus* NCDC118	Probiotics
Peng and Guo (2015) [73]	Milk yoghurt	Soy	Single culture (LAB)	*St. thermophilus* S7906	Dairy yoghurt
Li et al. (2014) [26]	Milk yoghurt	Soy	Single or mixed culture (LAB)	*L. plantarum* 70810, *L. rhamnosus* 6005, DVS YC-X11 (*L. bulgaricus*, *St.* *thermophilus*)	Fermented cabbage, Chinese sour soup, dairy yoghurt
Hickisch et al. (2016a) [158]	Milk yoghurt	Lupin	Single culture (LAB)	*L. bulgaricus* DSM 20080, *St.* *thermophilus* DSM 20259, *L. acidophilus* DSM 20079, *L. casei* DSM 20011, *Lc. lactis* DSM 20384, *Lc. cremoris* DSM 20069, *Leu. cremoris* DSM 20200, *L. helveticus* DSM 20057, *L. perolens* DSM 12744, *B. bifidum* DSM 20239, *L.* *plantarum* TMW 1.460, *L. plantarum* TMW 1.1468, *L. fermentum* DSM 20391, *L. pontis* TMW 1.1086, *L.* *sanfranciscensis* DSM 20451, *W. cibaria* TMW 2.1333, *L. brevis* TMW 1.1326, *L. brevis* BGT L150, *L. amylolyticus* BGT TL3, *L. amylolyticus* BGT TL5, *L. species* BGT TL11, *L. species* BGT TL13, *L.* *rossiae* BGT L1202, *Pe. pentosaceus* BGT B34, *Pe. pentosaceus* DSM 20336, *L.* *curvatus* TMW 1.624, *L. reuteri* DSM 20016, *L. buchneri* DSM 20057, *L. gasseri* DSM 20243, *B. lactis* DSM 10140	Fermented dairy, soy yoghurt, EPS-producers, probiotics, sourdough, slime producers, beer spoilage, antinutrient degradation
Luana et al. (2014) [53]	Milk yoghurt drink	Oat	Single culture (LAB)	*L. plantarum* (LP01, LP06, LP09, LP32, LP39, LP40, LP48, LP51), *L. casei* (LC10, LC11, LC03), *L. paracasei* (LPC02, LPC16)	Dairy probiotics
Coda et al. (2012) [121]	Milk yoghurt	Rice, soy, barley, wheat, emmer	Single, then mixed culture (LAB)	*L. plantarum* (6E, LB1, POM1, M6, PR1, 1LC5), *L. rossiae* LB5, *W. cibaria* (POM9, M1, 3XLC3), *Pe. pentosaceus* SWE5	Cereals, fruits, vegetables
Cheese					
Masia et al. (2022) [41]	Hard cheese	Pea protein isolate, olive oil	Mixed culture (LAB)	VEGA™ Harmony (*L. bulgaricus*, *St. thermophilus*, *L. acidophilus*, *L. paracasei*, *Bifidobacterium* spp.)	Dairy yoghurt
Ben-Harb et al. (2020) [159]	Off-flavour reduction in cheese analogue	Pea protein isolate	Single culture (Yeast, fungi, LAB)	*Co. casei* UCMA 3821, *Y. lipolytica* CLIB183, *Br. aurantiacum* ATCC 9174, *G. candidum* ATCC 204307, *Br. casei* CIP102111, *Glu. arilaitensis* CIP 108037, *Br. antiquum* CNRZ918, *C. catenulata* Exrfcom LD, *Sta. equorum* Mu2, *Ha. alvei* GB001, *L. rhamnosus* CNRZ212, *Lc. lactis* S3, *Leu. lactis* NCW1, *K. lactis* 3550, *D. hansenii* 304	Dairy cheese
Li et al. (2020) [28]	Soft cheese	Soy	Mixed culture (LAB, fungi)	*L. bulgaricus*, *St. thermophilus*, *G. candidum*	Dairy yoghurt, dairy cheese
Łopusiewicz et al. (2020a) [152]	Camembert cheese	Flaxseed	Mixed culture (LAB, fungi)	MST Cheese-Tek^®^ (*Lc. lactis*, *Lc.* *cremoris*, *St. thermophilus*), PC^®^ (*P.* *camemberti*), GEO^®^ (*G. candidum*)	Dairy cheese
Giri et al. (2018) [160]	Cream cheese spread	Soy	Single culture (LAB)	*L. casei* NCDC-017	Probiotics
Matias et al. (2014) [29]	Petit-suisse cheese	Soy	Mixed culture (LAB)	ABT-4 (includes *B. lactis* BB-12, *L.* *acidophilus* LA-5, *St. thermophilus*)	Dairy yoghurt
Li et al. (2013) [81]	Cream cheese spread	Soy	Mixed culture (LAB)	*L. acidophilus* NCFM™, *B. lactis* HOWARU™ Bifido	Probiotics
Kefir					
Yepez et al. (2019) [124]	Milk kefir	Oat, maize, barley	Mixed culture (LAB, yeast, AAB)	*L. plantarum* (M5MA1, M9MG6, M9MM1, M9MM4, M9Y2), *Leu. mesenteroides* (M9MG2b, T1M3), milk kefir, water kefir	Milk kefir, water kefir, fermented cereals, fermented tubers

^1^ Abbreviations of the microbial genus names are as follows: *Lactobacillus* (L.); *Penicillium* (P.); *Streptococcus* (St.); *Bifidobacterium* (B.); *Lactococcus* (Lc.); *Leuconostoc* (Leu.); *Geotrichum* (G.); *Corynebacterium* (Co.); *Yarrowia* (Y.); *Brevibacterium* (Br.); *Glutamicibacter* (Glu.); *Candida* (C.); *Staphylococcus* (Sta.); *Hafnia* (Ha.); *Kluyveromyces* (K.); *Debaryomyces* (D.); *Pediococcus* (Pe.); *Bacillus* (Bac.); *Weissella* (W.). ^2^ The complete scientific name of each strain is always provided where possible, e.g., *St. thermophilus*, *L. bulgaricus*. ^3^ Where a commercial starter culture is used, the product name is listed, followed by the specific cultures in parenthesis. * A probiotic *L. rhamnosus* strain was inoculated for viability tests during prolonged storage but was not deemed to have affected the organoleptic quality of the product.

## Data Availability

Not applicable.

## References

[1] Food and Agricultural Organisation of the United Nations Dairy and Dairy Products. https://www.fao.org/3/CB5332EN/Dairy.pdf.

[2] Tamang J.P., Shin D.H., Jung S.J., Chae S.W. (2016). Functional properties of microorganisms in fermented foods. Front. Microbiol..

[3] Mendly-Zambo Z., Powell L.J., Newman L.L. (2021). Dairy 3.0: Cellular agriculture and the future of milk. Food Cult. Soc..

[4] Cichonska P., Ziarno M. (2022). Legumes and legume-based beverages fermented with lactic acid bacteria as a potential carrier of probiotics and prebiotics. Microorganisms.

[5] Grand View Research Dairy Alternatives Market Share & Growth Report, 2021–2028. https://www.grandviewresearch.com/Industry-Analysis/Dairy-Alternatives-Market.

[6] Hartmann C., Hieke S., Taper C., Siegrist M. (2018). European consumer healthiness evaluation of ‘Free-from’ labelled food products. Food Qual. Prefer..

[7] Yadav D.N., Bansal S., Jaiswal A.K., Singh R. (2017). Plant Based Dairy Analogues: An Emerging Food. Agric. Res. Technol. Open Access J..

[8] Roselló-Soto E., Garcia C., Fessard A., Barba F.J., Munekata P.E.S., Lorenzo J.M., Remize F. (2019). Nutritional and microbiological quality of tiger nut tubers (*Cyperus esculentus*), derived plant-based and lactic fermented beverages. Fermentation.

[9] McClements D.J., Newman E., McClements I.F. (2019). Plant-based milks: A review of the science underpinning their design, fabrication, and performance. Compr. Rev. Food Sci. Food Saf..

[10] Jeske S., Zannini E., Arendt E.K. (2018). Past, present and future: The strength of plant-based dairy substitutes based on gluten-free raw materials. Food Res. Int..

[11] Kamath R., Basak S., Gokhale J. (2022). Recent trends in the development of healthy and functional cheese analogues—A review. LWT.

[12] Ferawati F., Hefni M., Östbring K., Witthöft C. (2021). The application of pulse flours in the development off plant-based cheese analogues: Proximate composition, color, and texture properties. Foods.

[13] Grossmann L., McClements D.J. (2021). The science of plant-based foods: Approaches to create nutritious and sustainable plant-based cheese analogs. Trends Food Sci. Technol..

[14] Ben-Harb S., Panouillé M., Huc-Mathis D., Moulin G., Saint-Eve A., Irlinger F., Bonnarme P., Michon C., Souchon I. (2018). The rheological and microstructural properties of pea, milk, mixed pea/milk gels and gelled emulsions designed by thermal, acid, and enzyme treatments. Food Hydrocoll..

[15] Sharma R., Mokhtari S., Jafari S.M., Sharma S. (2021). Barley-based probiotic food mixture: Health effects and future prospects. Crit. Rev. Food Sci. Nutr..

[16] Chen W., Zhu J., Niu H., Song Y., Zhang W., Chen H., Chen W. (2018). Composition and characteristics of Yam juice fermented by *Lactobacillus plantarum* and *Streptococcus thermophilus*. Int. J. Food Eng..

[17] Mäkinen O.E., Wanhalinna V., Zannini E., Arendt E.K. (2016). Foods for special dietary needs: Non-dairy plant-based milk substitutes and fermented dairy-type products. Crit. Rev. Food Sci. Nutr..

[18] Mefleh M., Pasqualone A., Caponio F., Faccia M. (2022). Legumes as basic ingredients in the production of dairy-free cheese alternatives: A review. J. Sci. Food Agric..

[19] Souza R.G.M., Gomes A.C., Naves M.M.V., Mota J.F. (2015). Nuts and legume seeds for cardiovascular risk reduction: Scientific evidence and mechanisms of action. Nutr. Rev..

[20] Petrova P., Petrov K. (2020). Lactic acid fermentation of cereals and pseudocereals: Ancient nutritional biotechnologies with modern applications. Nutrients.

[21] Chandrasekara A., Kumar T.J. (2016). Roots and tuber crops as functional foods: A review on phytochemical constituents and their potential health benefits. Int. J. Food Sci..

[22] Boeck T., Sahin A.W., Zannini E., Arendt E.K. (2021). Nutritional properties and health aspects of pulses and their use in plant-based yogurt alternatives. Compr. Rev. Food Sci. Food Saf..

[23] Vogelsang-O’Dwyer M., Zannini E., Arendt E.K. (2021). Production of pulse protein ingredients and their application in plant-based milk alternatives. Trends Food Sci. Technol..

[24] Lopes M., Pierrepont C., Duarte C.M., Filipe A., Medronho B., Sousa I. (2020). Legume beverages from chickpea and lupin as new milk alternatives. Foods.

[25] Jayarathna S., Priyashantha H., Johansson M., Vidanarachchi J.K., Jayawardana B.C., Liyanage R. (2021). Probiotic enriched fermented soy-gel as a vegan substitute for dairy yoghurt. J. Food Process. Preserv..

[26] Li C., Li W., Chen X., Feng M., Rui X., Jiang M., Dong M. (2014). Microbiological, physicochemical and rheological properties of fermented soymilk produced with exopolysaccharide (EPS) producing lactic acid bacteria strains. LWT.

[27] Pandey S.M., Mishra H.N. (2015). Optimization of the prebiotic & probiotic concentration and incubation temperature for the preparation of synbiotic soy yoghurt using response surface methodology. LWT.

[28] Li Y., Zhang X., Yang J.J., Ma X.Y., Jia X.D., Du P., Li A.L. (2020). Influence of the addition of *Geotrichum candidum* on the microbial, chemical, textural, and sensory features of soft soy cheese. J. Food Process. Preserv..

[29] Matias N.S., Bedani R., Castro I.A., Saad S.M.I. (2014). A probiotic soy-based innovative product as an alternative to petit-suisse cheese. LWT.

[30] Zhu Y.Y., Thakur K., Feng J.Y., Cai J.S., Zhang J.G., Hu F., Wei Z.J. (2020). B-vitamin enriched fermented soymilk: A novel strategy for soy-based functional foods development. Trends Food Sci. Technol..

[31] Inouye K., Shiihara M., Uno T., Takita T. (2002). Deodorization of soybean proteins by enzymatic and physicochemical treatments. J. Agric. Food Chem..

[32] Short E.C., Kinchla A.J., Nolden A.A. (2021). Plant-based cheeses: A systematic review of sensory evaluation studies and strategies to increase consumer acceptance. Foods.

[33] Horáčková Š., Mühlhansová A., Sluková M., Schulzová V., Plocková M. (2015). Fermentation of soymilk by yoghurt and bifidobacteria strains. Czech J. Food Sci..

[34] Feng S., Saw C.L., Lee Y.K., Huang D. (2008). Novel process of fermenting black soybean [*Glycine max* (L.) Merrill] yogurt with dramatically reduced flatulence-causing oligosaccharides but enriched soy phytoalexins. J. Agric. Food Chem..

[35] Meinlschmidt P., Ueberham E., Lehmann J., Schweiggert-Weisz U., Eisner P. (2016). Immunoreactivity, sensory and physicochemical properties of fermented soy protein isolate. Food Chem..

[36] Wang S., Chelikani V., Serventi L. (2018). Evaluation of chickpea as alternative to soy in plant-based beverages, fresh and fermented. LWT.

[37] Tangyu M., Fritz M., Aragao-Börner R., Ye L., Bogicevic B., Bolten C.J., Wittmann C. (2021). Genome-based selection and application of food-grade microbes for chickpea milk fermentation towards increased L-lysine content, elimination of indigestible sugars, and improved flavour. Microb. Cell Fact..

[38] Zhang T., Li Y., Miao M., Jiang B. (2011). Purification and characterisation of a new antioxidant peptide from chickpea (*Cicer arietium* L.) protein hydrolysates. Food Chem..

[39] Hickisch A., Bindl K., Vogel R.F., Toelstede S. (2016). Thermal treatment of lupin-based milk alternatives—Impact on lupin proteins and the network of respective lupin-based yogurt alternatives. Food Res. Int..

[40] Pontonio E., Raho S., Dingeo C., Centrone D., Carofiglio V.E., Rizzello C.G. (2020). Nutritional, functional, and technological characterization of a novel gluten- and lactose-free yogurt-style snack produced with selected lactic acid bacteria and *Leguminosae* flours. Front. Microbiol..

[41] Masiá C., Jensen P.E., Petersen I.L., Buldo P. (2022). Design of a functional pea protein matrix for fermented plant-based cheese. Foods.

[42] Zhang C., Hua Y., Li X., Kong X., Chen Y. (2020). Key volatile off-flavor compounds in peas (*Pisum sativum* L.) and their relations with the endogenous precursors and enzymes using soybean (*Glycine max*) as a reference. Food Chem..

[43] Davis J.P., Dean L.L. (2016). Peanut composition, flavor and nutrition. Peanuts: Genetics, Processing, and Utilization.

[44] Valero-Cases E., Cerdá-Bernad D., Pastor J.J., Frutos M.J. (2020). Non-dairy fermented beverages as potential carriers to ensure probiotics, prebiotics, and bioactive compounds arrival to the gut and their health benefits. Nutrients.

[45] Silva A.R.A., Silva M.M.N., Ribeiro B.D. (2020). Health issues and technological aspects of plant-based alternative milk. Food Res. Int..

[46] Demïr H., Simsek M., Yıldırım G. (2021). Effect of oat milk pasteurization type on the characteristics of yogurt. LWT.

[47] Paul A.A., Kumar S., Kumar V., Sharma R. (2020). Milk Analog: Plant based alternatives to conventional milk, production, potential and health concerns. Crit. Rev. Food Sci. Nutr..

[48] Lorusso A., Coda R., Montemurro M., Rizzello C.G. (2018). Use of selected lactic acid bacteria and quinoa flour for manufacturing novel yogurt-like beverages. Foods.

[49] Zannini E., Jeske S., Lynch K., Arendt E.K. (2018). Development of novel quinoa-based yoghurt fermented with dextran producer *Weissella cibaria* MG1. Int. J. Food Microbiol..

[50] King J.C., Blumberg J., Ingwersen L., Jenab M., Tucker K.L. (2008). Tree nuts and peanuts as components of a healthy diet. J. Nutr..

[51] Aydar E.F., Tutuncu S., Ozcelik B. (2020). Plant-based milk substitutes: Bioactive compounds, conventional and novel processes, bioavailability studies, and health effects. J. Funct. Foods.

[52] Qamar S., Manrique Y.J., Parekh H., Falconer J.R. (2020). Nuts, cereals, seeds and legumes proteins derived emulsifiers as a source of plant protein beverages: A review. Crit. Rev. Food Sci. Nutr..

[53] Luana N., Rossana C., Curiel J.A., Kaisa P., Marco G., Rizzello C.G. (2014). Manufacture and characterization of a yogurt-like beverage made with oat flakes fermented by selected lactic acid bacteria. Int. J. Food Microbiol..

[54] Briviba K., Gräf V., Walz E., Guamis B., Butz P. (2016). Ultra high pressure homogenization of almond milk: Physico-chemical and physiological effects. Food Chem..

[55] Bocker R., Silva E.K. (2022). Innovative technologies for manufacturing plant-based non-dairy alternative milk and their impact on nutritional, sensory and safety aspects. Future Foods.

[56] Fu W., Yano H. (2020). Development of “new” bread and cheese. Processes.

[57] Pachekrepapol U., Kokhuenkhan Y., Ongsawat J. (2021). Formulation of yogurt-like product from coconut milk and evaluation of physicochemical, rheological, and sensory properties. Int. J. Gastron. Food Sci..

[58] Saranraj P., Behera S.S., Ray R.C. (2019). Traditional foods from tropical root and tuber crops: Innovations and challenges. Innovations in Traditional Foods.

[59] Epping J., Laibach N. (2020). An underutilized orphan tuber crop—Chinese yam: A review. Planta.

[60] Nanbol K.K., Namo O.T.A. (2019). The contribution of root and tuber crops to food security: A review. J. Agric. Sci. Technol. B.

[61] Batista N.N., Ramos C.L., Pires J.F., Moreira S.I., Alves E., Dias D.R., Schwan R.F. (2019). Nondairy ice cream based on fermented yam (*Dioscorea* sp.). Food Sci. Nutr..

[62] Trim D.S., Marder R.C. (1995). Investigations of hydrocyclones for concentration of cassava milk. Starch-Stärke.

[63] Madsen S.K., Thulesen E.T., Mohammadifar M.A., Bang-Berthelsen C.H. (2021). Chufa drink: Potential in developing a new plant-based fermented dessert. Foods.

[64] Kizzie-Hayford N., Jaros D., Schneider Y., Rohm H. (2015). Characteristics of tiger nut milk: Effects of milling. Int. J. Food Sci. Technol..

[65] Tangyu M., Muller J., Bolten C.J., Wittmann C. (2019). Fermentation of plant-based milk alternatives for improved flavour and nutritional value. Appl. Microbiol. Biotechnol..

[66] Alcorta A., Porta A., Tárrega A., Alvarez M.D., Pilar Vaquero M. (2021). Foods for plant-based diets: Challenges and innovations. Foods.

[67] Ferawati F., Hefni M., Witthöft C. (2019). Flours from Swedish pulses: Effects of treatment on functional properties and nutrient content. Food Sci. Nutr..

[68] Munekata P.E.S., Domínguez R., Budaraju S., Roselló-Soto E., Barba F.J., Mallikarjunan K., Roohinejad S., Lorenzo J.M. (2020). Effect of innovative food processing technologies on the physicochemical and nutritional properties and quality of non-dairy plant-based beverages. Foods.

[69] Zaaboul F., Raza H., Cao C., Yuanfa L. (2019). The impact of roasting, high pressure homogenization and sterilization on peanut milk and its oil bodies. Food Chem..

[70] Ahmadian-Kouchaksaraei Z., Varidi M., Varidi M.J., Pourazarang H. (2014). Influence of processing conditions on the physicochemical and sensory properties of sesame milk: A novel nutritional beverage. LWT.

[71] Ma W., Zhang C., Kong X., Li X., Chen Y., Hua Y. (2021). Effect of pea milk preparation on the quality of non-dairy yoghurts. Food Biosci..

[72] Ghavidel R.A., Prakash J. (2007). The impact of germination and dehulling on nutrients, antinutrients, in vitro iron and calcium bioavailability and in vitro starch and protein digestibility of some legume seeds. LWT.

[73] Peng X., Guo S. (2015). Texture characteristics of soymilk gels formed by lactic fermentation: A comparison of soymilk prepared by blanching soybeans under different temperatures. Food Hydrocoll..

[74] Kaharso V.C., Muhoza B., Kong X., Hua Y., Zhang C. (2021). Quality improvement of soymilk as influenced by anaerobic grinding method and calcium addition. Food Biosci..

[75] Pineli L.L.O., Botelho R.B.A., Zandonadi R.P., Solorzano J.L., de Oliveira G.T., Reis C.E.G., Teixeira D.D.S. (2015). Low glycemic index and increased protein content in a novel quinoa milk. LWT.

[76] Vatansever S., Xu M., Magallanes-López A., Chen B., Hall C. (2021). Supercritical carbon dioxide + ethanol extraction to improve organoleptic attributes of pea flour with applications of sensory evaluation, HS-SPME-GC, and GC-olfactory. Processes.

[77] Guldiken B., Green R., Nickerson M.T. (2021). The impact of different adsorbents on flavour characteristics of a lentil protein isolate. Eur. Food Res. Technol..

[78] Wang Y., Guldiken B., Tulbek M., House J.D., Nickerson M. (2020). Impact of alcohol washing on the flavour profiles, functionality and protein quality of air classified pea protein enriched flour. Food Res. Int..

[79] Jiang Z.Q., Wang J., Stoddard F., Salovaara H., Sontag-Strohm T. (2020). Preparation and characterization of emulsion gels from whole faba bean flour. Foods.

[80] Park M.J., Lee S.Y. (2015). Quality characteristics of soy yogurt produced using proteases and mixed microbial consortia. J. Korean Soc. Appl. Biol. Chem..

[81] Li Q., Xia Y., Zhou L., Xie J. (2013). Evaluation of the rheological, textural, microstructural and sensory properties of soy cheese spreads. Food Bioprod. Process..

[82] Ogundipe O.O., Fasogbon B.M., Ogundipe F.O., Oredope O., Amaezenanbu R.U. (2021). Nutritional composition of non-dairy yogurt from sprouted tigernut tubers. J. Food Process. Preserv..

[83] Cáceres P.J., Peñas E., Martínez-villaluenga C., García-mora P., Frías J. (2019). Development of a multifunctional yogurt-like product from germinated brown rice. LWT.

[84] Eun C., Azizul H., Jin H., Lee H., Hun Y., Hee S., Lee Y., Cheol S., Cho K.M. (2018). Bioconversion of c-aminobutyric acid and isoflavone contents during the fermentation of high-protein soy powder yogurt with *Lactobacillus brevis*. Appl. Biol. Chem..

[85] Yang M., Li L. (2010). Physicochemical, textural and sensory characteristics of probiotic soy yogurt prepared from germinated soybean. Food Technol. Biotechnol..

[86] Hong P., Tung L., Diep T., Thi N., Nguyen A., Tran D., Thi T., Tran M., Trung T. (2021). Evaluation of physicochemical properties of soymilk prepared from germinated soybean. J. Food Process. Preserv..

[87] Levy R., Okun Z., Shpigelman A. (2022). Utilizing high-pressure homogenization for the production of fermented plant-protein yogurt alternatives with low and high oil content using potato protein isolate as a model. Innov. Food Sci. Emerg. Technol..

[88] Demirkesen I., Vilgis T.A., Mert B. (2018). Effect of microfluidization on the microstructure and physical properties of a novel yoghurt formulation. J. Food Eng..

[89] Ferragut V., Cruz N.S., Trujillo A., Guamis B., Capellas M. (2009). Physical characteristics during storage of soy yogurt made from ultra-high pressure homogenized soymilk. J. Food Eng..

[90] Xia X., Dai Y., Wu H., Liu X., Wang Y., Cao J., Zhou J. (2019). Effects of pressure and multiple passes on the physicochemical and microbial characteristics of lupin-based beverage treated with high-pressure homogenization. J. Food Process. Preserv..

[91] Jeske S., Bez J., Arendt E.K., Zannini E. (2019). Formation, stability, and sensory characteristics of a lentil-based milk substitute as affected by homogenisation and pasteurisation. Eur. Food Res. Technol..

[92] Mu Q., Su H., Zhou Q., Xiao S., Zhu L., Xu X., Pan S., Hu H. (2022). Effect of ultrasound on functional properties, flavor characteristics, and storage stability of soybean milk. Food Chem..

[93] Lu X., Chen J., Zheng M., Guo J., Qi J., Chen Y., Miao S., Zheng B. (2019). Effect of high-intensity ultrasound irradiation on the stability and structural features of coconut-grain milk composite systems utilizing maize kernels and starch with different amylose contents. Ultrason. Sonochem..

[94] Abdullah Z., Taip F.S., Mustapa Kamal S.M., Abdul Rahman R.Z. (2018). Effect of sodium caseinate concentration and sonication amplitude on the stability and physical characteristics of homogenized coconut milk. J. Food Process. Preserv..

[95] Wang C., Yin H., Zhao Y., Zheng Y., Xu X., Yue J. (2021). Optimization of high hydrostatic pressure treatments on soybean protein isolate to improve its functionality and evaluation of its application in yogurt. Foods.

[96] Sim S.Y.J., Srv A., Chiang J.H., Henry C.J. (2021). Plant proteins for future foods: A roadmap. Foods.

[97] Dhakal S., Liu C., Zhang Y., Roux K.H., Sathe S.K., Balasubramaniam V.M. (2014). Effect of high pressure processing on the immunoreactivity of almond milk. Food Res. Int..

[98] Manzoor M.F., Zeng X.A., Ahmad N., Ahmed Z., Rehman A., Aadil R.M., Roobab U., Siddique R., Rahaman A. (2020). Effect of pulsed electric field and thermal treatments on the bioactive compounds, enzymes, microbial, and physical stability of almond milk during storage. J. Food Process. Preserv..

[99] Li Y.Q., Tian W.L., Mo H.Z., Zhang Y.L., Zhao X.Z. (2013). Effects of pulsed electric field processing on quality characteristics and microbial inactivation of soymilk. Food Bioprocess Technol..

[100] Saldanha do Carmo C., Silventoinen P., Nordgård C.T., Poudroux C., Dessev T., Zobel H., Holtekjølen A.K., Draget K.I., Holopainen-Mantila U., Knutsen S.H. (2020). Is dehulling of peas and faba beans necessary prior to dry fractionation for the production of protein- and starch-rich fractions? Impact on physical properties, chemical composition and techno-functional properties. J. Food Eng..

[101] Kwok K.C., Niranjan K. (1995). Review: Effect of thermal processing on soymilk. Int. J. Food Sci. Technol..

[102] Guo X., McClements D.J., Chen J., He X., Liu W., Dai T., Liu C. (2021). The nutritional and physicochemical properties of whole corn slurry prepared by a novel industry-scale microfluidizer system. LWT.

[103] Giri S.K., Mangaraj S. (2012). Processing influences on composition and quality attributes of soymilk and its powder. Food Eng. Rev..

[104] Rinaldoni A.N., Palatnik D.R., Zaritzky N., Campderrós M.E. (2014). Soft cheese-like product development enriched with soy protein concentrates. LWT.

[105] Sethi S., Tyagi S.K., Anurag R.K. (2016). Plant-based milk alternatives an emerging segment of functional beverages: A review. J. Food Sci. Technol..

[106] Bansal S., Mangal M., Sharma S.K., Yadav D.N., Gupta R.K. (2016). Optimization of process conditions for developing yoghurt like probiotic product from peanut. LWT.

[107] Agrahar-Murugkar D., Bajpai-Dixit P., Kotwaliwale N. (2020). Rheological, nutritional, functional and sensory properties of millets and sprouted legume based beverages. J. Food Sci. Technol..

[108] Hu M., Du X., Liu G., Zhang S., Wu H., Li Y. (2021). Germination improves the functional properties of soybean and enhances soymilk quality. Int. J. Food Sci. Technol..

[109] Mäkinen O.E., Uniacke-Lowe T., O’Mahony J.A., Arendt E.K. (2015). Physicochemical and acid gelation properties of commercial UHT-treated plant-based milk substitutes and lactose free bovine milk. Food Chem..

[110] Zamora A., Guamis B. (2015). Opportunities for ultra-high-pressure homogenisation (UHPH) for the food industry. Food Eng. Rev..

[111] Gul O., Saricaoglu F.T., Mortas M., Atalar I., Yazici F. (2017). Effect of high pressure homogenization (HPH) on microstructure and rheological properties of hazelnut milk. Innov. Food Sci. Emerg. Technol..

[112] Cruz N., Capellas M., Hernández M., Trujillo A.J., Guamis B., Ferragut V. (2007). Ultra high pressure homogenization of soymilk: Microbiological, physicochemical and microstructural characteristics. Food Res. Int..

[113] Cruz N.S., Capellas M., Jaramillo D.P., Trujillo A.J., Guamis B., Ferragut V. (2009). Soymilk treated by ultra high-pressure homogenization: Acid coagulation properties and characteristics of a soy-yogurt product. Food Hydrocoll..

[114] Yang M., Li N., Tong L., Fan B., Wang L., Wang F., Liu L. (2021). Comparison of physicochemical properties and volatile flavor compounds of pea protein and mung bean protein-based yogurt. LWT.

[115] Poliseli-Scopel F.H., Hernández-Herrero M., Guamis B., Ferragut V. (2014). Sterilization and aseptic packaging of soymilk treated by ultra high pressure homogenization. Innov. Food Sci. Emerg. Technol..

[116] Pérez-González M., Gallardo-Chacón J.J., Valencia-Flores D., Ferragut V. (2015). Optimization of a headspace SPME GC–MS methodology for the analysis of processed almond beverages. Food Anal. Methods.

[117] Iswarin S.J., Permadi B. (2012). Coconut milk’s fat breaking by means of ultrasound. Int. J. Basic Appl. Sci. IJBAS-IJENS.

[118] Gharibzahedi S.M.T., Smith B. (2020). The functional modification of legume proteins by ultrasonication: A review. Trends Food Sci. Technol..

[119] Manassero C.A., Vaudagna S.R., Sancho A.M., Añón M.C., Speroni F. (2016). Combined high hydrostatic pressure and thermal treatments fully inactivate trypsin inhibitors and lipoxygenase and improve protein solubility and physical stability of calcium-added soymilk. Innov. Food Sci. Emerg. Technol..

[120] Liu G.C., Gong X.Y., Jiang Y., Piao Y.Q., Fang X., Huang Z.J., Tian D. (2019). Research and application of mass spectrometry with low energy electron impact ionization source. Chin. J. Anal. Chem..

[121] Coda R., Lanera A., Trani A., Gobbetti M., Di Cagno R. (2012). Yogurt-like beverages made of a mixture of cereals, soy and grape must: Microbiology, texture, nutritional and sensory properties. Int. J. Food Microbiol..

[122] Adejuyitan J.A., Olanipekun B.F., Moyinwin O.A. (2014). Production and evaluation of cheese-like product from the blend of soy milk and coconut milk. Arch. Appl. Sci. Res..

[123] Oyeyinka A.T., Odukoya J.O., Adebayo Y.S. (2019). Nutritional composition and consumer acceptability of cheese analog from soy and cashew nut milk. J. Food Process. Preserv..

[124] Yépez A., Russo P., Spano G., Khomenko I., Biasioli F., Capozzi V., Aznar R. (2019). In situ riboflavin fortification of different kefir-like cereal-based beverages using selected Andean LAB strains. Food Microbiol..

[125] Rasika D.M.D., Vidanarachchi J.K., Luiz S.F., Azeredo D.R.P., Cruz A.G., Ranadheera C.S. (2021). Probiotic delivery through non-dairy plant-based food matrices. Agriculture.

[126] Rasika D.M., Vidanarachchi J.K., Rocha R.S., Balthazar C.F., Cruz A.G., Sant’Ana A.S., Ranadheera C.S. (2021). Plant-based milk substitutes as emerging probiotic carriers. Curr. Opin. Food Sci..

[127] Aryana K.J., Olson D.W. (2017). A 100-Year Review: Yogurt and other cultured dairy products. J. Dairy Sci..

[128] Hickisch A. (2020). Impact of Lactic Fermentation and Thermal Treatments on the Texture of a Lupin-Based Yogurt Alternative. Ph.D. Thesis.

[129] Gänzle M.G. (2015). Lactic metabolism revisited: Metabolism of lactic acid bacteria in food fermentations and food spoilage. Curr. Opin. Food Sci..

[130] Macori G., Cotter P.D. (2018). Novel insights into the microbiology of fermented dairy foods. Curr. Opin. Biotechnol..

[131] Zheng J., Wittouck S., Salvetti E., Franz C.M.A.P., Harris H.M.B., Mattarelli P., O’toole P.W., Pot B., Vandamme P., Walter J. (2020). A taxonomic note on the genus *Lactobacillus*: Description of 23 novel genera, emended description of the genus *Lactobacillus beijerinck* 1901, and union of *Lactobacillaceae* and *Leuconostocaceae*. Int. J. Syst. Evol. Microbiol..

[132] Oren A., Arahal D.R., Rosselló-Móra R., Sutcliffe I.C., Moore E.R.B. (2021). Preparing a revision of the international code of nomenclature of prokaryotes. Int. J. Syst. Evol. Microbiol..

[133] Bintsis T. (2018). Lactic acid bacteria as starter cultures: An update in their metabolism and genetics. AIMS Microbiol..

[134] Licandro H., Ho P.H., Nguyen T.K.C., Petchkongkaew A., Van Nguyen H., Chu-Ky S., Nguyen T.V.A., Lorn D., Waché Y. (2020). How fermentation by lactic acid bacteria can address safety issues in legumes food products?. Food Control.

[135] Bourdichon F., Casaregola S., Farrokh C., Frisvad J.C., Gerds M.L., Hammes W.P., Harnett J., Huys G., Laulund S., Ouwehand A. (2012). Food fermentations: Microorganisms with technological beneficial use. Int. J. Food Microbiol..

[136] Nejati F., Junne S., Neubauer P. (2020). A big world in small grain: A review of natural milk Kefir starters. Microorganisms.

[137] Irlinger F., Helinck S., Jany J.L. (2017). Secondary and Adjunct Cultures.

[138] Bintsis T. (2021). Yeasts in different types of cheese. AIMS Microbiol..

[139] Rohm H., Eliskases-Lechner F., Bräuer M. (1992). Diversity of yeasts in selected dairy products. J. Appl. Bacteriol..

[140] Breuer U., Harms H. (2006). *Debaryomyces hansenii*—An extremophilic yeast with biotechnological potential. Yeast.

[141] Fröhlich-Wyder M.T., Arias-Roth E., Jakob E. (2019). Cheese yeasts. Yeast.

[142] Prado M.R., Blandón L.M., Vandenberghe L.P.S., Rodrigues C., Castro G.R., Thomaz-Soccol V., Soccol C.R. (2015). Milk kefir: Composition, microbial cultures, biological activities, and related products. Front. Microbiol..

[143] Büchl N.R., Seiler H. (2011). Yeasts and Molds: Yeasts in Milk and Dairy Products. Encyclopedia of Dairy Sciences.

[144] Shori A.B. (2012). Comparative study of chemical composition, isolation and identification of micro-flora in traditional fermented camel milk products: Gariss, Suusac, and Shubat. J. Saudi Soc. Agric. Sci..

[145] Wang S.Y., Chen H.C., Liu J.R., Lin Y.C., Chen M.J. (2008). Identification of yeasts and evaluation of their distribution in Taiwanese kefir and viili starters. J. Dairy Sci..

[146] Afzaal M., Saeed F., Anjum F., Waris N., Husaain M., Ikram A., Ateeq H., Muhammad Anjum F., Suleria H. (2021). Nutritional and ethnomedicinal scenario of koumiss: A concurrent review. Food Sci. Nutr..

[147] Bhattacharya I., Yan S., Yadav J.S.S., Tyagi R.D., Surampalli R.Y. (2013). *Saccharomyces unisporus*: Biotechnological Potential and Present Status. Compr. Rev. Food Sci. Food Saf..

[148] Singh P.K., Shah N.P. (2017). Other Fermented Dairy Products: Kefir and Koumiss.

[149] Rattray F.P., O’Connell M.J. (2011). Kefir: Fermented Milk. Encyclopedia of Dairy Sciences.

[150] Khrundin D., Ponomarev V., Yunusov E., Ezhkova G. (2021). The use of plant proteins in the technology of fermented dairy-free products. IOP Conf. Ser. Earth Environ. Sci..

[151] Aydar A.Y., Mataracı C.E., Sağlam T.B. (2021). Development and modeling of a novel plant-based yoghurt produced by Jerusalem artichoke and almond milk using l-optimal mixture design. J. Food Meas. Charact..

[152] Łopusiewicz Ł., Drozłowska E., Tarnowiecka-Kuca A., Bartkowiak A., Mazurkiewicz-Zapałowicz K., Salachna P. (2020). Biotransformation of flaxseed oil cake into bioactive camembert-analogue using lactic acid bacteria, *Penicillium camemberti* and *Geotrichum candidum*. Microorganisms.

[153] Raikos V., Juskaite L., Vas F., Hayes H.E. (2020). Physicochemical properties, texture, and probiotic survivability of oat-based yogurt using aquafaba as a gelling agent. Food Sci. Nutr..

[154] Brückner-Gühmann M., Banovic M., Drusch S. (2019). Towards an increased plant protein intake: Rheological properties, sensory perception and consumer acceptability of lactic acid fermented, oat-based gels. Food Hydrocoll..

[155] Ani E., Amove J., Igbabul B. (2018). Physicochemical, microbiological, sensory properties and storage stability of plant-based yoghurt produced from bambaranut, soybean and *Moringa oleifera* seed milks. Am. J. Food Nutr..

[156] Ermiş E., Güneş R., Zent İ., Çağlar M.Y., Yılmaz M.T. (2018). Characterization of hazelnut milk fermented by *Lactobacillus delbrueckii* subsp.*bulgaricus* and *Streptococcus thermophilus*. Gida/J. Food.

[157] Falade K.O., Ogundele O.M., Ogunshe A.O., Fayemi O.E., Ocloo F.C.K. (2015). Physico-chemical, sensory and microbiological characteristics of plain yoghurt from bambara groundnut (*Vigna subterranea*) and soybeans (*Glycine max*). J. Food Sci. Technol..

[158] Hickisch A., Beer R., Vogel R.F., Toelstede S. (2016). Influence of lupin-based milk alternative heat treatment and exopolysaccharide-producing lactic acid bacteria on the physical characteristics of lupin-based yogurt alternatives. Food Res. Int..

[159] Ben-Harb S., Irlinger F., Saint-Eve A., Panouillé M., Souchon I., Bonnarme P. (2020). Versatility of microbial consortia and sensory properties induced by the composition of different milk and pea protein-based gels. LWT.

[160] Giri S.K., Tripathi M.K., Kotwaliwale N. (2018). Effect of composition and storage time on some physico-chemical and rheological properties of probiotic soy-cheese spread. J. Food Sci. Technol..

[161] Łopusiewicz Ł., Drozłowska E., Siedlecka P., Mężyńska M., Bartkowiak A. (2020). Preparation and characterization of novel flaxseed oil cake yogurt-like plant milk fortified with inulin. J. Food Nutr. Res..

[162] Lynch K.M., Zannini E., Coffey A., Arendt E.K. (2018). Lactic acid bacteria exopolysaccharides in foods and beverages: Isolation, properties, characterization, and health benefits. Annu. Rev. Food Sci. Technol..

[163] Zannini E., Waters D.M., Coffey A., Arendt E.K. (2016). Production, properties, and industrial food application of lactic acid bacteria-derived exopolysaccharides. Appl. Microbiol. Biotechnol..

[164] Blaya J., Barzideh Z., LaPointe G. (2018). Symposium review: Interaction of starter cultures and nonstarter lactic acid bacteria in the cheese environment. J. Dairy Sci..

[165] Jeewanthi R.K.C., Paik H.D. (2018). Modifications of nutritional, structural, and sensory characteristics of non-dairy soy cheese analogs to improve their quality attributes. J. Food Sci. Technol..

[166] Awad S., Hassan A.N., Muthukumarappan K. (2005). Application of exopolysaccharide-producing cultures in reduced-fat Cheddar cheese: Texture and melting properties. J. Dairy Sci..

[167] Zhang L., Li X., Ren H., Liu L., Ma L., Li M., Bi W. (2015). Impact of using exopolysaccharides (EPS)-producing strain on qualities of half-fat cheddar cheese. Int. J. Food Prop..

[168] Lynch K.M., Wilkinson S., Daenen L., Arendt E.K. (2021). An update on water kefir: Microbiology, composition and production. Int. J. Food Microbiol..

[169] Egea M.B., Santos D.C.D., Oliveira Filho J.G.D., Ores J.D.C., Takeuchi K.P., Lemes A.C. (2020). A review of nondairy kefir products: Their characteristics and potential human health benefits. Crit. Rev. Food Sci. Nutr..

[170] Cui X.H., Chen S.J., Wang Y., Han J.R. (2013). Fermentation conditions of walnut milk beverage inoculated with kefir grains. LWT.

[171] Abdolmaleki F., Mazaheri Assadi M., Akbarirad H. (2015). Assessment of beverages made from milk, soya milk and whey using Iranian kefir starter culture. Int. J. Dairy Technol..

[172] Łopusiewicz Ł., Drozłowska E., Siedlecka P., Mężyńska M., Bartkowiak A., Sienkiewicz M., Zielińska-Bliźniewska H., Kwiatkowski P. (2019). Development, characterization, and bioactivity of non-dairy kefir-like fermented beverage based on flaxseed oil cake. Foods.

[173] Sabokbar N., Moosavi-Nasab M., Khodaiyan F. (2015). Preparation and characterization of an apple juice and whey based novel beverage fermented using kefir grains. Food Sci. Biotechnol..

[174] Nedele A.K., Bär A., Mayer N., Schiebelbein R., Zhang Y. (2022). Characterization of cheesy odor formed during fermentation of soy drink with *Agrocybe aegerita*. Food Chem..

[175] Shi Y., Singh A., Kitts D.D., Pratap-Singh A. (2021). Lactic acid fermentation: A novel approach to eliminate unpleasant aroma in pea protein isolates. LWT.

[176] Youssef C.E., Bonnarme P., Fraud S., Péron A.C., Helinck S., Landaud S. (2020). Sensory improvement of a pea protein-based product using microbial co-cultures of lactic acid bacteria and yeasts. Foods.

[177] Arteaga V.G., Leffler S., Muranyi I., Eisner P., Schweiggert-Weisz U. (2021). Sensory profile, functional properties and molecular weight distribution of fermented pea protein isolate. Curr. Res. Food Sci..

[178] Peyer L.C., Zannini E., Arendt E.K. (2016). Lactic acid bacteria as sensory biomodulators for fermented cereal-based beverages. Trends Food Sci. Technol..

